# Deciphering the possible role of RNA-helicase genes mechanism in response to abiotic stresses in rapeseed (*Brassica napus* L.)

**DOI:** 10.1186/s12870-024-04893-0

**Published:** 2024-03-20

**Authors:** Bahareh Fatahi, Karim Sorkheh, Adriano Sofo

**Affiliations:** 1 Department of Production Engineering and Plant Genetics, Faculty of Agriculture, Shahid Chamran University of Ahvaz, P.O. Box 61355/144, Ahvaz, Iran; 2 Department of European and Mediterranean Cultures, Architecture, Environment, Cultural Heritage (DiCEM), Università degli Studi della Basilicata, Via Lanera 20, 75100 Matera, MT Italy

**Keywords:** RNA helicase (RH), Abiotic stresses, Regulation mechanism, Rapeseed, Survey genome analysis, Gene expression, Interaction network

## Abstract

**Background:**

Plants mediate several defense mechanisms to withstand abiotic stresses. Several gene families respond to stress as well as multiple transcription factors to minimize abiotic stresses without minimizing their effects on performance potential. RNA helicase (RH) is one of the foremost critical gene families that can play an influential role in tolerating abiotic stresses in plants. However, little knowledge is present about this protein family in rapeseed (canola). Here, we performed a comprehensive survey analysis of the RH protein family in rapeseed (*Brassica napus* L.).

**Results:**

A total of 133 BnRHs genes have been discovered in this study. By phylogenetic analysis, RHs genes were divided into one main group and a subgroup. Examination of the chromosomal position of the identified genes showed that most of the genes (27%) were located on chromosome 3. All 133 identified sequences contained the main DEXDC domain, the HELICC domain, and a number of sub-domains. The results of biological process studies showed that about 17% of the proteins acted as RHs, 22% as ATP binding, and 14% as mRNA binding. Each part of the conserved motifs, communication network, and three-dimensional structure of the proteins were examined separately. The results showed that the RWC in leaf tissue decreased with higher levels of drought stress and in both root and leaf tissues sodium concentration was increased upon increased levels of salt stress treatments. The proline content were found to be increased in leaf and root with the increased level of stress treatment. Finally, the expression patterns of eight selected RHs genes that have been exposed to drought, salinity, cold, heat and cadmium stresses were investigated by qPCR. The results showed the effect of genes under stress. Examination of gene expression in the Hayola #4815 cultivar showed that all primers except primer #79 had less expression in both leaves and roots than the control level.

**Conclusions:**

New finding from the study have been presented new insights for better understanding the function and possible mechanism of RH in response to abiotic stress in rapeseed.

**Supplementary Information:**

The online version contains supplementary material available at 10.1186/s12870-024-04893-0.

## Introduction

 Rapeseed (*Brassica napus* L.) belongs to the Brassicaceae family and is a product of temperate and temperate coastal areas. The high adaptation of oil seed to different climatic and geographical conditions will cause the cultivation of this plant will expand in many areas in the future. Climate change affects the need to develop commercial products in stressful conditions while improving the quality and performance of plants increases the need for this issue. Therefore, understanding crop physiology and plant response to stress allows to develop new varieties with traits of stress-tolerant [1, 2]. As a result, this plant will face different conditions that can include various environmental stresses [3].

Plants, during their growing season, are constantly faced with complex biotic and abiotic stresses such as drought, salinity, heat, cold and heavy metals, each of which directly or indirectly affects the final potential of plant performance. Moreover, plants have shown a series of self-regulatory mechanisms in response to abiotic stresses that lead to tolerance to such adverse environmental conditions. Therefore, several gene families respond to stress and multiple transcription factors to minimize the abiotic stresses on reduced performance potential [4, 5].

Abiotic stresses are the major constraints on the agricultural production in arid and semi-arid regions and include the negative effect of environmental factors such as light, temperature, soil salinity, heavy metals, etc., in plants [6]. In Iran, among abiotic stress, drought is the biggest obstacle to rapeseed growth and production [7]. Lack of water reduces cell swelling, stomatal conduction, and photosynthesis, and ultimately impairs growth and crop production. The harmful effect of salinity on plants is the result of osmotic stress and also the special effect of ions [8]. Since most crops are sensitive to salinity, improving salinity tolerance plays a very important role in maintaining agricultural productivity. Plants stimulate stress-related genes, proteins and metabolite accumulation to cope with the adverse consequences of salinity [9]. Due to the combined effects of osmotic potential and reduction in specific ions’ toxicity, high soil salt levels may significantly reduce seed germination and seedling growth. The tolerance of plants to low temperatures is different and by changing the degree of tolerance that can be caused by the cold acclimation phenomenon in plants [10]. Manifestation of cold stress injury in susceptible plants is different from cold tolerant plants and occurs within 2–3 days of exposure [11]. Phenotypic symptoms of cold stress in plants include reduced leaf expansion, reduced biomass, chlorosis and necrosis [12, 13]. Thermal stress is a serious limitation in the environment for plant growth and a major limiting factor for agricultural productivity and it has been shown that most performance components are affected by thermal stress [14, 15]. Biostimulants such as melatonin (MET) have a multifunctional role that acts as a “defense molecule” to protect plants against the harmful effects of temperature stress with MET treatment by improving several defense mechanisms and improving plant growth and temperature tolerance [9]. Exposure to high-level Cd can lead to a significant accumulation of Cd in foods [16]. In most plant species, cadmium is collected in the root, and a small amount are transmitted to the leaves. Efficient and economical remediation of polluted urban and agricultural lands is an urgent need for sustainable agricultural development prospects. Various methods such as biological, chemical, and physical have been used to remove heavy metal pollutants from soil. In wheat, Cd tolerance is associated with high activity of antioxidant enzymes, photosynthesis rate and hormone concentration [17, 18].

RNA-Helicase (RH) genes is one of the most important gene families that can play an effective role in tolerating abiotic stresses in plants and belongs to a classification of promoter molecular proteins found in yeast, animals, and plants. This family of genes is energy-dependent and is responsible for the cleavage of DNA or RNA [19]. On the other hand, RNA helicases represent a large family of proteins that are involved in modulating RNA structure and therefore affect splicing, RNA synthesis, amplification, editing, initiation of translation, rRNA processing, ribosome synthesis, mRNA stabilization and degradation. In general, all helicases generally have at least three common biochemical properties, including nucleic acid binding, NTP / dNTP binding, and degradation and have DNA or RNA-dependent NTPase activity. These enzymes are usually compatible with other enzymes or proteins in the metabolic activity of DNA [20].

Many RHs are essential for survival and play a key regulatory role in the cell. All eukaryotic RHs belong to SF1 and SF2 and energy-dependent enzymes that are responsible for breaking down DNA or RNA. These enzymes play a significant role in gene regulation, and expression. RHs are involved in the modification and synthesis of ribonucleotides, RNPs, pre-mRNA binding and cellular signaling against viral infection by activating interferons and cytokines through the phosphorylation of transcription factors [19]. DEAD-box helicase is one of the most important subfamilies of RHs. eIF4A is the first protein to be shown to be a helicase that was able to open RNA strands during virus replication [21]. Linder et al. [22] showed that many proteins share a consolidation of the protected helicase motif. DEAD-box helicases are found in prokaryotes as well as in eukaryotes. Some DEAD-box helicases are involved in nucleotide binding in both ATP-dependent and ATP-independent methods. Studies in *Arabidopsis* have shown that a lack of the eIF4A gene in the DEAD-box family reduces lateral root formation, delayed flowering, and abnormal seed growth, suggesting that eIF4A plays a crucial role in plant growth. Studies have shown that the DEAD-box *AtRH7* gene causes cold tolerance in *Arabidopsis*. Although there are many amino acids, such as glycine betaine and proline that show drought tolerance, there are also some genes in the DEAD box that help plants tolerate drought stress. In sorghum, DEAD-box protein HVD1 of *Hordeum vulgare*, an ATP-dependent helicase RNA, has been reported in response to salinity stress. It has recently been shown that a helicase DEAD-box RNA gene from *Arabidopsis* (*AtRH7*) is involved in the mechanism of cold tolerance [23]. High temperatures cause dehydration, membrane damage and enzyme inhibition in plants. However, there are some genes provide heat tolerance to plants. Recently, a DEAD-B-box gene called TOGR1 (heat-resistant growth) has been reported to improve crop productivity by increasing expression at high-temperature conditions in rice and also maintains rRNA homeostasis under heat stress [24].

Oil seed plants are threatened by several environmental stresses. This study has been the first to survey RHs family genes in *Brassica napus*; we know little knowledge of their functions in growth and responses to environmental stresses. Thus, a genome-wide survey of BnRH genes will help to understand molecular complex mechanisms. In the current study, the bioinformatics approaches were used to survey genomic RH gene family members of rapeseed, including genes number, location of chromosomes, relationships of phylogenetic, structural features and expression analysis of selected genes in responses to abiotic stress, cross-talk and networking of RHs members in *Brassica napus* in responses to environmental stresses conditions. This funding lays the groundwork for the functional characterization, and possible role of RHs mechanisms in response to abiotic stress in rapeseed.

## Materials and methods

This study consists of bioinformatics, laboratory and greenhouse survey each of which is discussed in detail. To identify the RH gene family in rapeseed, databases were first analyzed to identify these genes using bioinformatics databases and software. Afterward, practical experiments were performed on rapeseed to confirm their possible role mechanisms in response to drought, salinity, heat, cold, and cadmium in rapeseed. This research was performed in a bioinformatics, greenhouse, and laboratory experimental in the Faculty of Agriculture of Shahid Chamran University of Ahvaz, Iran.

### Bioinformatics approaches

#### Identification of RHs family protein sequences in rapeseed genome

Protein sequences, CDSs, and other rapeseed plant information were taken from the NCBI database (http://www.ncbi.nlm.nih) for further analysis. Using the decrease redundancy tool (http://web.expasy.org/decrease_redundancy/), the non-redundant sequences of candidate RHs were obtained. Then, after removing the redundant sequence of protein, RHs domains were examined by Pfam (http://www.sanger.ac.uk/Software/Pfam/) database (the accession number; PF00271, Helicase conserved C-terminal domain). The protein sequences of RNA- helicase were used to perform local BlastP search (with e-value < 1e-10) against *Brassica napus* L., then the sequences of candidate were used to HMM profile through hmmbuild program and so searched to obtain all of BnRHs genes members. After that all of the candidate genes were submitted to NCBI search.

#### Alignment of sequence and phylogenetic analysis of BnRHs in rapeseed

Alignment and drawing of phylogenetic tree protein sequences were obtained using ClustalX software, and their phylogenetic tree was drawn based on the neighbor-joining method and bootstrap with 1000 times repetition by MEGA ver.7 software.

##### Determination of physical properties and chromosomal distribution of BnRHs proteins

The Protparam tool from expasy database (http://www.expasy.com/protparam) was used to determine the physical and chemical properties of RHs proteins, including the number of amino acids in each protein, molecular weight, and isoelectric point (*p*I). To determine the distribution of RNA-helicase genes on 38 rapeseed chromosomes, information about the start and end points of each gene and the chromosome number of each gene were extracted from Expasy (http://www.expasy.org/tools/blast) and EnsemblePlant (http://plants.ensembl.org/Multi/Tools/Blast) databases and then the position of each gene on the chromosome was mapped using Mapchart ver.2.3 software.

#### Predicting the subcellular localization, motif, and gene structure of BnRHs proteins

The Softberry database (http://www.linux1.softberry.com/) was used to predict where each of the RHs proteins is located and active. In order to predict the domain and motif analysis, the protein sequences of BnRHs were put through using MEME (http://meme.sdsc.edu/meme/website/intro.html), according to the following parameters: number of maximum motifs = 15; widths of motifs optimum = 200 amino acid residues. The CDS and genome sequence of RHs genes of rapeseed were downloaded in FASTA format, and using the online tool Gene Structure Display Server (GSDS) (http://www.gsds.cbi.pku.edu.cn/) was used to investigate the variation of structure and number of exon/intron of member of BnRHs gene family [25].

#### Investigation of the co-expression gene network BnRHs proteins

The STRING (https://string-db.org/) database was used to identify the communication network and the interactions between proteins and functional physical interactions. In this way, each RHs protein in rapeseed was placed in front of a protein in this base with the *Arabidopsis* model plant. Then the communication network and the proteins involved in this communication path were displayed.

#### GO- analysis and promoter regions of BnRHs proteins

In order to predict the annotation and functional protein sequences of BnRHs blast2Go (http://www.blast2go.com) software was used [26]. The output of go-ontology is classified into molecular function, cellular components, and biological processes. In order to identify *Cis-*regulatory elements in the promoter regions of RHs genes and their possible role in the extent and expression of these genes in the development of resistance to abiotic stresses, the 2000 bp region upstream of each gene using the PLACE database (http://www.dna.affrc.go.jp/PLACE) was analyzed.

#### Determination of homology model in BnRHs sequences

Protein sequences of BnRHs were queried at the protein Data Bank (PDB) with BLASTP to recognize similarity sequences and the best 3D –structure [27]. 3D protein structure of BnRHs were estimated using ‘normal’ modeling of mode85 into Phyre2 server (Protein Homology/AnalogY Recognition Engine; http://www.sbg.bio.ic.ac.uk/ phyre2) [28] according to > 90% confidence level and > 80% similarity.

#### Detection of gene duplications and Ka/Ks analysis in BnRHs

Identification of tandem or segmental status among RHs sequences detected in rapeseed is from the distance between the target genes. Two genes are considered paralogous, if the genes with e-value equal to 1e -10 are 80% similar. The genes with more than 5 Mb, are considered as segmental duplication [29]. Tandem duplications were illustrated as contiguous genes of the same subfamily [30]. The Ka/Ks analysis of BnRHs genes in rapeseed was estimated using DnaSPver.5.10.1 software [31].

#### Determination of divergence of orthology BnRHs genes in rapeseed with *Arabidopsis *and tomato

PGDBj (Plant Genome DataBase Japan) was used to determine orthologous genes between rapeseed and *Arabidopsis*, and tomato. The time of duplication (million years ago or MYO) and divergence were calculated using the following formula.

$$\lambda=\mathrm{Ks}\;/\;2\lambda\;\left(\lambda\;=\;6.5\;\times\;10^{-9}\right)\;=\;\mathrm T$$

The ratio of dissimilarity (Ka) versus similar substitution (Ks) is a good measure of the selection pressure following doubling [32].

### Greenhouse experiments

Two cultivars of Hayola#50 and #4815 are one of the hybrid varieties of rapeseed that are suitable for cultivation in plain areas were selected. Hayola variety has different types, among which Hayola #50 is late compared to Hayola 4815. For germination kinetics measurements, two cultivars of Hayola#50 and #4815, at least three biological replicates (100 seeds per replicate) were imbibed on moistened filter paper in a petri dish for germination filled with 50 mL distilled water and germinated in a growth chamber a constant temperature of 20 °C and 50–75% relative humidity. The number of germinated seeds and the emergence of the first buds were evaluated.

### Plant cultivation conditions and treatment of abiotic stresses

*Brassica napus* L. seeds of Hayola#50 and Hayola#4815 were planted in pots with a specific volume (2 kg), and the plants were grown in natural conditions without stress in the greenhouse. The required seeds were placed in such a way that first, the bottom of the pots was drilled to create proper drainage and, a small amount of pebbles was poured into each pot. Then, for each pot, a mixture of pot soil and field soil was prepared. After seedling growth in 5 to 7 leaf stages, drought, salinity, heat, cold and heavy metals (cadmium) stresses were applied to the seedlings as follows, leaf and root samples frozen in liquid nitrogen and stored in -80 °C until RNA extraction. However, before RNA extraction, the soil around the roots was thoroughly washed with distilled water and finally treated with DEPES water so as not to interfere with the extraction process. This experiment was performed in a Randomized Complete Block (RCB) design with three replications.

### Drought stress

The seedlings with fully expanded leaves were subjected to three optimal irrigation regimes (field capacity (planting capacity, FC = 100%), 30 and 60% for 21 days, and then leaf and root samples were collected and stored rapidly in frozen liquid nitrogen until RNA extraction [33]. The relative water content (RWC) of leaf water [34] was calculated using the following formula.

$$\mathrm{RWC}\%=\lbrack(\mathrm{WF}-\mathrm{WD})/(\mathrm{WS}-\mathrm{WD})\rbrack\times100$$

### Salinity stress

To impose saline stress, six-week-old plantlets were exposed to saline treatments including 50, 100, and 200 mM NaCl and after two weeks of stress, sampling of leaf and roots was performed [35]. The ratio of Na^+^/K^+^ content of samples were also measured by a flame photometer (Jenway, PFP-7, Cole-Parmer Ltd, Stone, Staffordshire, UK) as a criterion for measuring different levels of salinity stress.

### Heat stress

To apply heat stress, the seedlings with 5 to 7 expanded leaves were subjected to 25 ± 1 °C (control) and 37 ± 1 °C (heat stress) in a growth chamber with 70% relative humidity and then at intervals of 1, 4, 6, and 12 h from the leaf and roots of stressed plants, sampling was performed [36, 37].

### Cold stress

The seedlings with leaf expanded were placed at 4 °C in the cold room, and then at intervals of 3, 6, 9, 12, and 24 h, the leaf and root tissue was sampled [38].

### Cd stress

Cd stress was performed on seedlings with 5 to 7 leaves (six-week-old) with a solution containing concentrations of 0 (control), 200, 400, 600, and 800 µM cadmium chloride were taken daily, and after two weeks, samples were taken from different organs (roots and leaves), then stored in frozen liquid nitrogen and stored at -80 ° C until analysis [39, 40].

### Determination of free proline content

Free proline levels for each abiotic stress, as an indicator, were measured using the method of Bates et al. [41] as follows: First, 500 mg of fresh plant tissue was ground in 10 mL of 3% sulfosalicylic acid, and a homogeneous mixture was prepared. The mixture was filtered using Whatman #20 filter paper, and then 2 ml of the filtered extract was mixed with 2 mg of ninhydrin reagent and 2 mL of acetic acid and boiled for 1 h at 100 °C. Next, 4 ml of toluene was added to the mixture, and the tubes were shaken well. Holding the tubes in place for 15–20 min, two completely separate layers formed in them. Absorption of a certain amount of this dye at 520 nm using a spectrophotometer and the amount of proline is determined as mgg^−1^FW was calculated.

### Experimental of laboratory

Before performing the relevant experiments, all required laboratory equipment was sterilized with an autoclave at 120 ° C and one atmospheric pressure for 15 min.

### Total RNA extraction and cDNA synthesis

RNA extraction from leaf and root seedlings was performed using the extraction kit (column RNA extraction kit, Dena Zist Asia, Cat. No.: S-1020-1; www.denazist.ir) according to the manufacture of instructions, and then treated with DNase I (SinaClone Ltd., Iran) to eliminate remaining genomic DNA. The enzyme is inactivated according to the instructions of the kit SinaClone ltd. Iran. The quality of the RNA samples was appraised by electrophoresis separation on a 0.8% agarose gel and biophotometer spectrophotometer (Eppendorf, Germany). Total cDNA synthesis was performed using an Eassy cDNA synthesis kit (ParsTous, Ltd. Cat: A101161, Iran; www.parstous.com/) according to the manufacture instructions of the kit.

### Design of primers and expression analysis of BnRHs genes by qPCR

The primers required for this study were designed using Primer3 online software (www.primer3.com). Eight specific primer pairs of BnRHs genes based on 3D protein model among the identified genes with above 80% homology selected and were synthesized by Pishgam (www.pishgambc.com) Biotechnology (Table 1). Finally, the primers were diluted and stored at -20 °C by the formula provided by the manufacturer. In order to investigate the expression pattern of BnRHs genes in response to drought, salinity, heat, cold and heavy metals (cadmium), a real-time chain reaction was used. The qPCR was used with SYBR Green I (Mastermix ParsTous, Iran) and monitored with the Master Cycler System (ABI, Biosystem, USA). The qPCR reactions were completed using the conditions listed as follows; 5 min at 95 °C, 40 cycles for 10 s at 95 °C for denaturation, 15s at 60 °C for annealing (according to T_m_ of each primer), and 30 s at 72 °C for elongation. The melting curve analysis was performed by denaturation at 95 °C for 15 s, and then the temperature was gradually increased from 60 to 95 °C using the default setting. These reactions were performed for three biological repeats. Relative expression were obtained using the comparative Ct (2^−ΔΔCt^) method [42], also *β*-*actin#7* was used as a reference gene to normalize expression values. Negative control was used to detect contamination in qPCR. All steps of qPCR were performed entirely on ice.

**Table 1:** Sequences of primers used in qRT-PCR

Oligo Name	Oligo Sequence 5’--> 3’	Product size(bp)
Bn_RH022_F	CGATTCTGGGCATGGATGTG	206
Bn_RH022_R	CACGCTCACTTTGGTATCCG	
Bn_RH025_F	CGATTCTGGGCATGGATGTG	206
Bn_RH025_R	CACGCTCACTTTGGTATCCG	
Bn_RH026_F	CGATTCTGGGCATGGATGTG	206
Bn_RH026_R	CACGCTCACTTTGGTATCCG	
Bn_RH033_F	CGGATACCAAAGTGAGCGTG	249
Bn_RH033_R	CCTGTTTATCATGCGGGGTC	
Bn_RH070_F	TATGAAAGAACAGCAGCGCG	159
Bn_RH070_R	CATACGCCACCACCACTTTC	
Bn_RH081_F	GGTGAGCCCGATTAGCAAAG	224
Bn_RH081_R	GCACCAGATCATCAATGCCC	
Bn_RH113_F	CGGCCTGGATGTGAAAGATG	166
Bn_RH113_R	GCTCACCAGTTCTTTCGCAA	
Bn_RH079_F	GGTGAGCCCGATTAGCAAAG	224
Bn_RH079_R	GCACCAGATCATCAATGCCC	
Actin-7_F	ACCCGGTTCTTCTCACTGAG	
Actin-7_R	AGGATAGCGTGAGGAAGAGC	

### Statistical data analysis

The statistical analysis was performed using SAS software (version 9.0) at a 5% level of significance. The heat map of gene expression of BnRHs genes was illustrated using HemI_1.6_alpha_win32_86.

## Results

### Identification of BnRHs protein family in rapeseed

In order to study the BnRHs protein family, the desired protein sequences were extracted from the NCBI site and examined. All the members of BnRHs identified in rapeseed for domain RHs protein based on Pfam and SMART verified. We discovered 133 candidate BnRHs genes in the genome of rapeseed that are listed in Table 2, which includes basic information such as isoelectric point (*p*I), length of proteins, molecular weight (Mw), and annotation at NCBI. The length of protein (aa) encoded by BnRH varied from 294 (*BnRH#100*) to 2188 (*BnRH#059*). The estimated molecular weight of BnRHs candidate genes was distributed in ranged from 33.21 (*BnRH#100*) to 246.67 (*BnRH#117*) kDa. The predicted isoelectric points (*p*I) of the BnRHs genes candidate were between 5.08 (*BnRH#108*) to 9.88 (*BnRH#037*).

**Table 2:** Position, protein length and chromosomal location, protein length, isoelectric point and molecular weight of RHs genes identified in rapeseed. Chromosomal location 0 means unknown specific gene location on *B. napus* genome

Generic Name	Accession number	Locus Tag(Chr)	Start position(bp)	End position(bp)	Sequence Length	Location	PI	MW (kDa)	Protein Length(aa)
BnRH-001	XP_013641459.1	3	341531	342724	1193	Nuclear	9.33	65.90923	583
BnRH-002	XP_013642084.1	9	208559	211653	3094	Nuclear	8.68	81.68811	723
BnRH-003	XP_013642191.1	7	209724	211043	1319	None	5.3	131.7769	1167
BnRH-004	XP_013642261.1	5	451258	452136	878	Nuclear	5.63	134.0163	1172
BnRH-005	XP_013642587.1	3	1494980	1498357	3377	Cytoplasmic	6.12	150.1436	1341
BnRH-006	XP_013642882.1	4	134261	136180	1919	None	5.89	88.75401	784
BnRH-007	XP_013643004.1	6	233177	235993	2816	None	9.13	79.96606	711
BnRH-008	XP_013643558.1	4	541313	545395	4082	None	7.85	144.5641	1300
BnRH-009	XP_013643805.1	7	1364611	1367118	2507	Nuclear	8.67	80.94812	750
BnRH-010	XP_013644733.1	6	420376	423140	2764	Nuclear	6.53	133.6476	1171
BnRH-011	XP_013644857.1	7	28076	33816	5740	Cytoplasmic	8.76	127.7522	1135
BnRH-012	XP_013645934.1	1	146054	149326	3272	Nuclear	9.08	86.0119	762
BnRH-013	XP_013647906.1	5	306071	307747	1676	Nuclear	9.21	63.50123	602
BnRH-014	XP_013650345.1	7	3053762	3055444	1682	Nuclear	6.29	65.76943	588
BnRH-015	XP_013652066.1	7	3053762	3055444	1682	Nuclear	6.29	65.90809	591
BnRH-016	XP_013653825.1	9	77998	79107	1109	None	9.49	87.73748	779
BnRH-017	XP_013655152.1	8	526	2841	2315	Nuclear	8.34	72.76071	644
BnRH-018	XP_013656160.1	10	19064	20718	1654	Nuclear	8.32	74.95494	671
BnRH-019	XP_013661111.1	9	374305	376452	2147	Nuclear	9.31	63.30078	584
BnRH-020	XP_013661582.1	9	155278	156587	1309	None	9.36	87.86369	779
BnRH-021	XP_013661716.1	9	295276	301212	5936	None	6	162.6938	1454
BnRH-022	XP_013661881.1	3	169756	172283	2527	Nuclear	5.4	48.32136	427
BnRH-023	XP_013664768.1	1	433518	436114	2596	Mitochondrial	8.83	64.1609	571
BnRH-024	XP_013666728.1	10	69125	70432	1307	Nuclear	8.84	84.10664	744
BnRH-025	XP_013668087.1	3	169756	172283	2527	Nuclear	5.39	48.29331	427
BnRH-026	XP_013668088.1	3	169756	172283	2527	Nuclear	5.46	48.32046	427
BnRH-027	XP_013668464.1	1	168717	171031	2314	None	8.85	67.42601	597
BnRH-028	XP_013669288.2	1	11494	12584	1090	Nuclear	8.55	120.6485	1073
BnRH-029	XP_013669290.2	1	11494	12584	1090	Nuclear	7.84	88.11516	777
BnRH-030	XP_013670607.1	1	1241658	1243017	1359	Nuclear	8.67	69.30655	624
BnRH-031	XP_013670873.2	0	84287	85342	1055	None	6.35	136.4882	1207
BnRH-032	XP_013672528.1	1	10280	12945	2665	Nuclear	6.12	111.9179	988
BnRH-033	XP_013673115.2	3	169756	172000	2244	Nuclear	6.28	39.07519	344
BnRH-034	XP_013676146.1	2	491779	494667	2888	Nuclear	8.69	66.58459	594
BnRH-035	XP_013678233.1	0	108823	111283	2460	Cytoplasmic	9.07	50.28709	448
BnRH-036	XP_013679045.1	3	169756	172283	2527	Nuclear	5.34	48.37537	427
BnRH-037	XP_013681379.1	7	3665918	3669619	3701	Nuclear	9.88	93.88803	852
BnRH-038	XP_013682891.1	5	1247232	1248941	1709	Nuclear	9.1	65.01979	576
BnRH-039	XP_013683473.2	3	677191	678483	1292	Nuclear	9.7	79.01425	686
BnRH-040	XP_013683593.2	3	1311916	1314072	2156	Cytoplasmic	8.4	111.1316	1002
BnRH-041	XP_013683613.1	2	591701	593387	1686	Nuclear	6.66	77.72024	691
BnRH-042	XP_022552831.1	2	185221	188261	3040	None	8.69	127.592	1128
BnRH-043	XP_022553276.1	6	267642	269840	2198	Nuclear	5.29	55.11382	491
BnRH-044	XP_022553334.1	3	1134199	1134990	791	None	6.3	49.18817	433
BnRH-045	XP_022554310.1	3	169756	172000	2244	None	6.83	39.08528	344
BnRH-046	XP_022554728.1	2	805550	806829	1279	None	9.52	72.33683	678
BnRH-047	XP_022554729.1	2	460676	462247	1571	None	9.45	71.83726	674
BnRH-048	XP_022554897.1	3	896000	898879	2879	Nuclear	7.56	82.93802	728
BnRH-049	XP_022554898.1	3	896000	898879	2879	Nuclear	6.96	82.52553	725
BnRH-050	XP_022554899.1	3	896794	898879	2085	Nuclear	5.67	60.86225	545
BnRH-051	XP_022554900.1	3	896803	898879	2076	Nuclear	5.67	60.69204	543
BnRH-052	XP_022554991.1	3	229605	232399	2794	None	10	68.58063	621
BnRH-053	XP_022555160.1	3	1138324	1139205	881	Nuclear	6	49.31524	433
BnRH-054	XP_022555294.1	3	655559	656710	1151	Nuclear	9.04	65.05093	576
BnRH-055	XP_022555295.1	3	655559	656710	1151	Nuclear	9.04	65.05093	576
BnRH-056	XP_022555315.1	3	314013	315563	1550	None	8.81	112.5296	1023
BnRH-057	XP_022555636.1	3	153299	156485	3186	Nuclear	8.34	78.63318	701
BnRH-058	XP_022555637.1	3	153299	156485	3186	Nuclear	8.34	78.63318	701
BnRH-059	XP_022556805.1	3	590717	593629	2912	Nuclear	5.46	248.6525	2188
BnRH-060	XP_022556806.1	3	590717	593629	2912	Nuclear	5.46	248.6525	2188
BnRH-061	XP_022556806.1	3	590717	593629	2912	Nuclear	5.46	248.6525	2188
BnRH-062	XP_022556805.1	3	590717	593629	2912	Nuclear	5.46	248.6525	2188
BnRH-063	XP_022557328.1	4	267662	269741	2079	Cytoplasmic	8.38	58.2996	510
BnRH-064	XP_022559130.1	2	2633	5008	2375	Nuclear	5.59	104.6118	931
BnRH-065	XP_022559133.1	2	2633	5008	2375	Nuclear	5.49	97.93944	872
BnRH-066	XP_022559296.1	5	1524365	1525636	1271	Nuclear	8.81	39.11425	350
BnRH-067	XP_022559343.1	5	1522842	1525636	2794	Nuclear	8.4	118.7235	1040
BnRH-068	XP_022559620.1	5	1978	4329	2351	Nuclear	6.4	71.68714	630
BnRH-069	XP_022560593.1	7	3053762	3054853	1091	Nuclear	6.29	65.7895	587
BnRH-070	XP_022560750.1	7	2090122	2092347	2225	Cytoplasmic	8.41	68.29908	640
BnRH-071	XP_022561763.1	2	1036267	1040371	4104	Nuclear	5.92	115.022	1022
BnRH-072	XP_022562044.1	2	2633	5008	2375	Nuclear	5.59	104.6118	931
BnRH-073	XP_022562045.1	2	2633	5008	2375	Nuclear	5.59	104.6118	931
BnRH-074	XP_022562046.1	2	2633	5008	2375	Nuclear	5.59	104.6118	931
BnRH-075	XP_022562047.1	2	2633	5008	2375	Nuclear	5.59	104.6118	931
BnRH-076	XP_022562048.1	2	2633	5008	2375	Nuclear	5.59	104.6118	931
BnRH-077	XP_022562049.1	2	2633	5008	2375	Nuclear	5.59	104.6118	931
BnRH-078	XP_022562050.1	2	2633	5008	2375	Nuclear	5.59	104.6118	931
BnRH-079	XP_022563558.1	8	240734	242806	2072	Nuclear	5.85	61.04151	546
BnRH-080	XP_022563559.1	8	240722	242806	2084	Nuclear	5.71	60.13258	537
BnRH-081	XP_022563560.1	8	241092	242806	1714	Nuclear	5.71	58.93916	526
BnRH-082	XP_022563577.1	8	462969	464615	1646	Mitochondrial	9.38	60.27747	550
BnRH-083	XP_022563578.1	8	462969	464615	1646	Mitochondrial	9.38	60.27747	550
BnRH-084	XP_022563579.1	8	462969	464615	1646	Mitochondrial	9.38	60.27747	550
BnRH-085	XP_022563985.1	8	22190	25263	3073	Nuclear	5.58	89.22268	791
BnRH-086	XP_022565518.1	9	145949	147948	1999	Cytoplasmic	9.38	37.58496	331
BnRH-087	XP_022565745.1	9	1343959	1346143	2184	Nuclear	9.42	71.41801	656
BnRH-088	XP_022566116.1	9	300950	304250	3300	Cytoplasmic	7.63	76.66016	681
BnRH-089	XP_022566256.1	5	451258	452136	878	Nuclear	5.92	133.4735	1165
BnRH-090	XP_022566256.1	5	451258	452136	878	Nuclear	5.92	133.4735	1165
BnRH-091	XP_022566753.1	3	498725	502353	3628	Chloroplast	6.72	197.595	1757
BnRH-092	XP_022568937.1	1	94903	97128	2225	Nuclear	8.57	102.6114	893
BnRH-093	XP_022569691.1	5	1248996	1250125	1129	Nuclear	9	63.4981	562
BnRH-094	XP_022570372.1	5	1247232	1248941	1709	Nuclear	9.1	65.01979	576
BnRH-095	XP_022570373.1	5	1248996	1250125	1129	Nuclear	9	63.4981	562
BnRH-096	XP_022570388.1	1	472691	474941	2250	Nuclear	6.08	42.55468	384
BnRH-097	XP_022572398.1	3	524321	526803	2482	Cytoplasmic	9.13	51.15402	454
BnRH-098	XP_022572540.1	1	10280	12945	2665	Nuclear	7.17	94.10989	826
BnRH-099	XP_022572585.1	3	1103675	1105270	1595	Nuclear	5.34	61.69911	554
BnRH-100	XP_022572983.1	3	1134199	1134990	791	Nuclear	5.31	33.21523	294
BnRH-101	XP_022574002.1	3	677191	678483	1292	Nuclear	9.62	77.20646	673
BnRH-102	XP_022574172.1	4	68703	70360	1657	Cytoplasmic	7.19	66.41617	614
BnRH-103	XP_022574173.1	4	68703	70360	1657	Cytoplasmic	7.19	66.41617	614
BnRH-104	XP_022575207.1	5	1747505	1749879	2374	None	5.93	148.0964	1330
BnRH-105	XP_022575571.1	5	200412	202272	1860	Nuclear	6.14	110.9465	988
BnRH-106	XP_022575572.1	5	200412	202272	1860	Nuclear	6.14	110.9465	988
BnRH-107	XP_022575574.1	5	200412	202272	1860	Nuclear	6.14	110.9465	988
BnRH-108	XP_022575630.1	1	43353	45903	2550	None	5.08	130.0163	1184
BnRH-109	XP_022575632.1	1	43353	45903	2550	None	5.57	121.4964	1104
BnRH-110	XP_022576263.1	6	1333558	1338750	5192	None	9.16	132.7857	1180
BnRH-111	XP_022576264.1	6	1333558	1338750	5192	None	9.16	132.7857	1180
BnRH-112	XP_022576283.1	9	1343959	1345272	1313	Nuclear	9.14	49.32652	455
BnRH-113	XP_013740565.1	3	729532	732365	2833	Nuclear	9.1	55.19874	496
BnRH-114	XP_013725991.1	1	472691	474941	2250	Nuclear	8.27	77.47191	694
BnRH-115	XP_013679449.1	2	1146345	1150499	4154	Nuclear	6.15	141.0769	1248
BnRH-116	XP_013679452.1	2	1146345	1150499	4154	Nuclear	6.15	141.148	1248
BnRH-117	XP_013695003.1	3	590714	593629	2915	Nuclear	5.45	248.6708	2186
BnRH-118	XP_013731451.1	5	11939	13951	2012	Nuclear	8.67	59.79658	540
BnRH-119	XP_013734891.1	3	1103675	1106068	2393	Nuclear	6.36	80.89966	711
BnRH-120	XP_013737451.1	7	1364611	1367004	2393	Chloroplast	6.68	80.68474	748
BnRH-121	XP_013742020.1	5	11924	14339	2415	Nuclear	8.86	59.29709	536
BnRH-122	XP_013742643.1	5	9817	11860	2043	Nuclear	5.57	118.7134	1042
BnRH-123	XP_013747521.1	5	2024226	2028422	4196	None	5.78	113.9999	1011
BnRH-124	XP_013747522.1	5	2024226	2028422	4196	None	5.78	113.6244	1008
BnRH-125	XP_013747524.1	4	312782	314721	1939	Nuclear	5.7	79.90518	732
BnRH-126	XP_013749451.1	8	239870	242806	2936	Nuclear	7.54	81.78601	718
BnRH-127	XP_013750006.1	6	267642	269831	2189	Nuclear	5.29	55.05872	490
BnRH-128	XP_013750644.1	5	223480	225386	1906	None	8.44	83.14401	744
BnRH-129	XP_013642191.1	7	209724	211043	1319	None	5.25	123.864	1097
BnRH-130	XP_013642261.1	5	451258	452136	878	Nuclear	5.6	125.4121	1102
BnRH-131	XP_013642263.1	5	451258	452136	878	Nuclear	5.63	134.0433	1172
BnRH-132	XP_013643303.1	3	341531	342724	1193	Nuclear	9.27	67.31187	594
BnRH-133	XP_013643558.1	4	541313	545395	4082	None	7.18	136.7354	1230

#### Subcellular localization and domains of BnRHs genes family

**Fig. 1:**
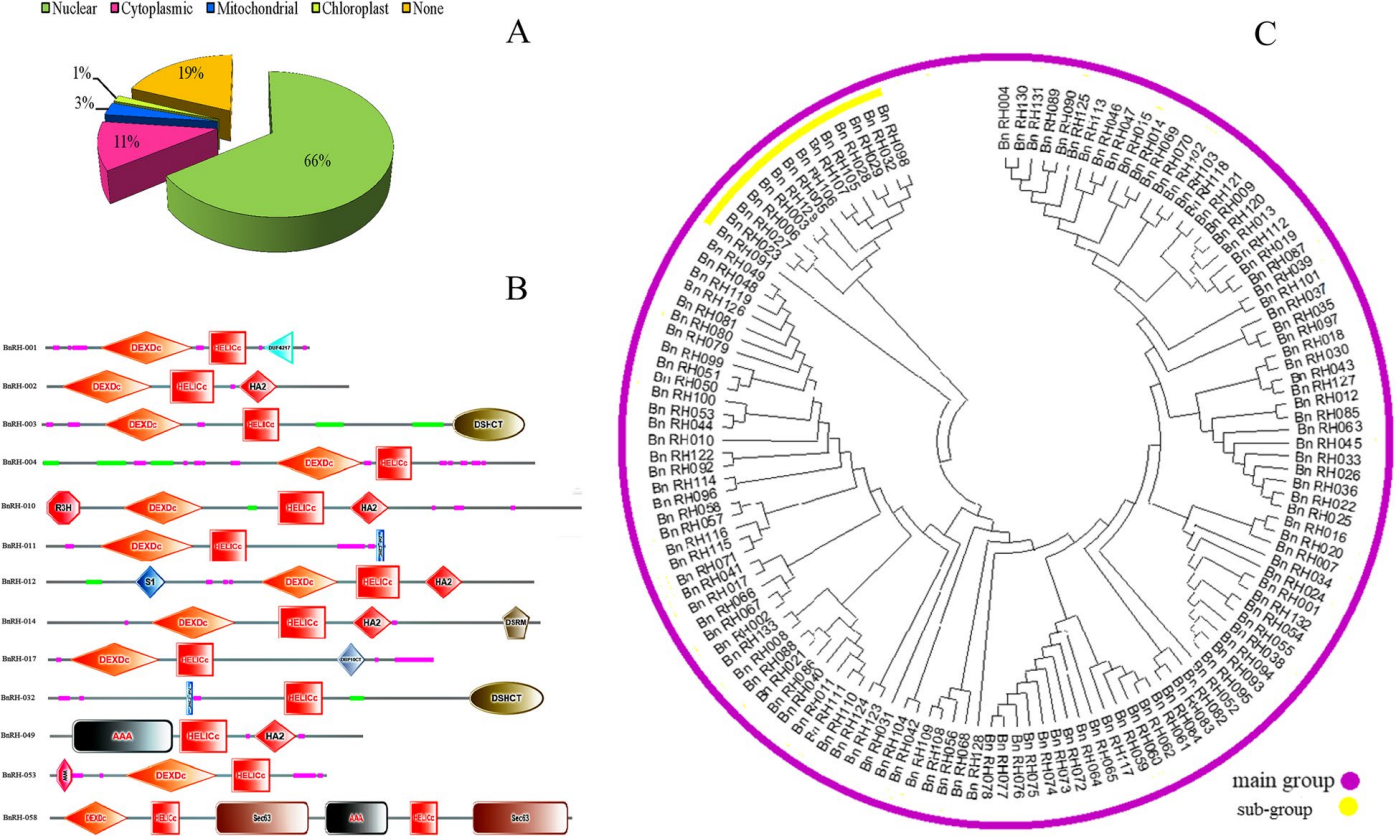
Predicting the distribution frequency and cellular location (**A**) and domains (**B**) identified and phylogenetic tree (**C**) analyzed BnRHs sequences in rapeseed. Total proteins were used to construct the neighbor-joining method and bootstrap with 1000 times repetition. BnRH proteins divided to two groups that marked with different colors main group (purple color) and sub group (yellow color)

 The survey within 133 BnRHs proteins in the Softberry database illustrated in Fig. 1A as follows: 90 proteins are located in the nucleus (66%), 15 proteins (11%) in the cytoplasm, 4 proteins (3%) in the mitochondria, 2 proteins (1%) in the chloroplast, and 26 proteins (19%) did not have a definite position.

 SMART databases were used in the obtained sequences to examine the relevant domains. All 133 identified sequences had the main DEXDC domain and HELICC domain and sub-domains including: DUF 4218, HA2, R3H, S1, DSRM, DSHCT, and Sect. 63 (Fig. 1B). Some sequences, including *BnRH#049* and *BnRH#058*, had a poly-A tail. Therefore, considering the diversity of these domains, it can be concluded that there is diversity in the function of biological structures.

### Phylogeny tree, gene structure, and motif analysis of BnRHs proteins in rapeseed

**Fig. 2:**
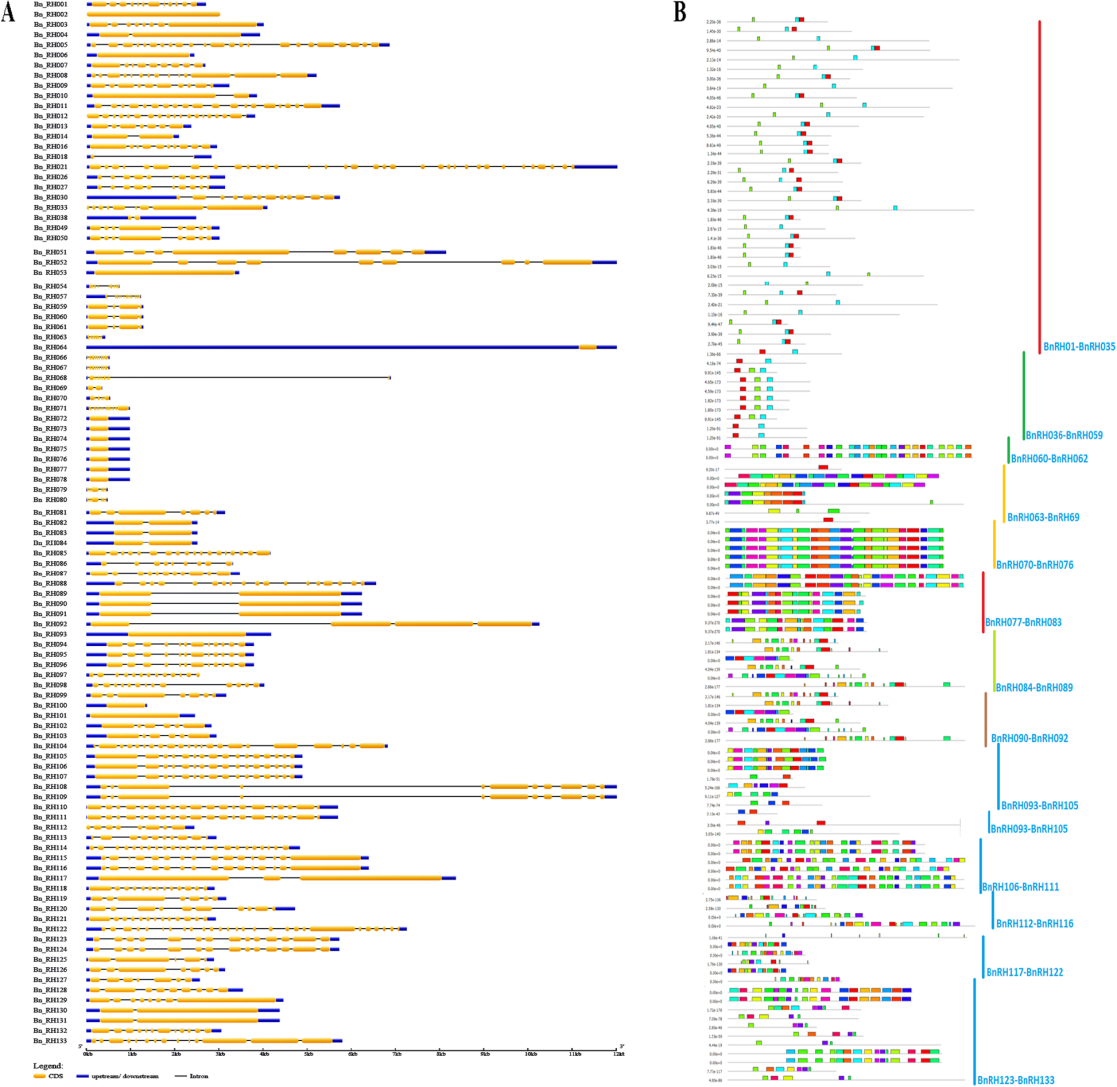
Structural distribution of introns / exons length are displayed, yellow boxes represents exons, the lines with black color represents introns, and the boxes with blue colors represents upstream and downstream (**A**); and number of distribution available motifs (main and sub) of the total BnRH proteins. The different color represents maximum number of motif = 15 (**B**) in the BnRHs protein sequence in rapeseed

 The phylogenetic tree of 133 proteins was drawn by the neighbor-joining method with 1000 bootstraps. Accordingly, all sequences were divided into a main group (purple) and a subgroup (yellow) (Fig. 1C). Analysis of the gene structure in the family protein gene using the GSDS database showed that the number of intron/exon regions of these genes varied from 0 introns (*BnRH#002*, *BnRH#064*) to 33 intron regions (*BnRH#021*) (Fig. 2A).

Figure 2B showed MEME results of 133 BnRHs of rapeseed. These results illustrated conserved domains among proteins’ sequence and identified 100 conserved motifs. 100 motifs were identified, named motif 1 to motif 100, respectively. A common domain and motif in proteins indicates a similar function and causes a similar functional role and activity in proteins.

### 3D-homology and network analysis of BnRHs proteins

The 3D protein model was demonstrated based on the sequence similarity of the PDB database using BLASTP. Among the identified proteins, the structure of 8 proteins with homology above 80% was determined, and their three-dimensional model was displayed using Phyer2 databases (Additional file 1A).

In this study, the linkage analysis between BnRHs proteins and *Arabidopsis* was performed. MTR4, HEN2, EMB30, FAS4, and RH20 proteins in the gene network have established stronger links (Additional file 1B).

### Gene ontology

**Fig. 3:**
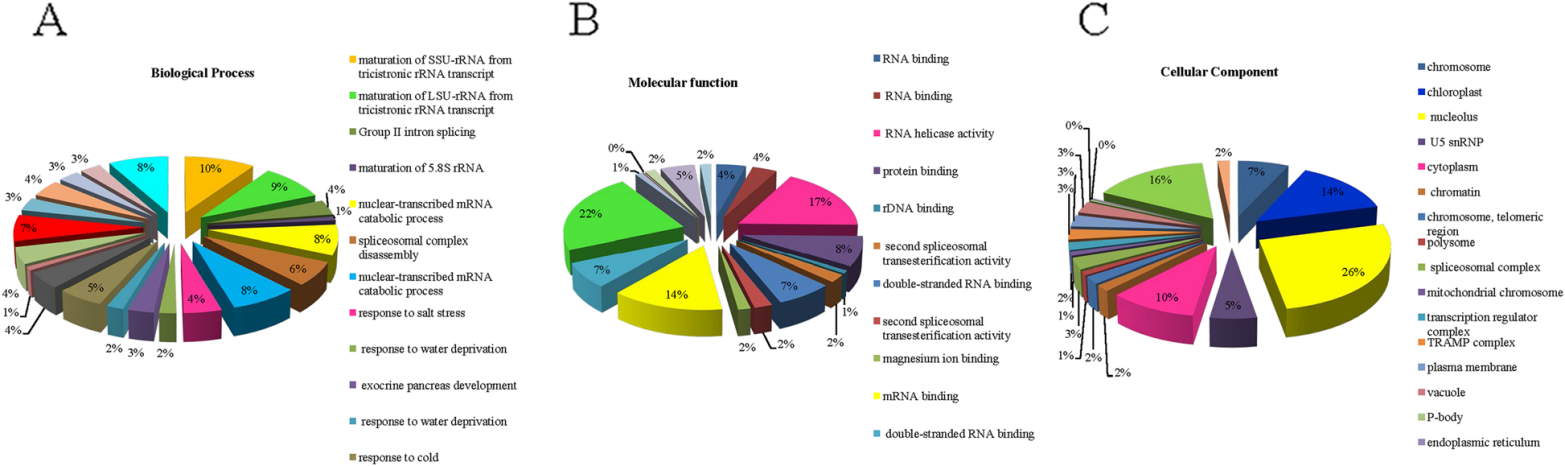
Distribution frequency and go- ontology of BnRNA-helicase sequences in rapeseed: (**A**) biological processes, (**B**) molecular function, (**C**) and cellular component of BnRNA-helicase sequences in rapeseed

 Blast2Go software was used to survey of Go annotation of BnRHs. The results showed that a main proportion of BnRHs played roles in binding, helicase activity, cellular component, and response to salt, water deprivation, cold, and cadmium (Cd). The results showed that about 10% of BnRHs proteins are involved in the processes related to the maturation of SSU-rRNA from tricistronic rRNA transcript and 8% in the process of catabolic nucleus mRNA transcription. In addition, about 17% of the proteins act as RNA helicase, 22% as ATP binding, and 14% as mRNA binding, and the highest activity in the nucleus (26%), Plasmodium (16%), and chloroplasts (14%), respectively, and the rest in other categories (Fig. 3A-C).

### Chromosomal distribution of BnRHs genes in rapeseed

**Fig. 4:**
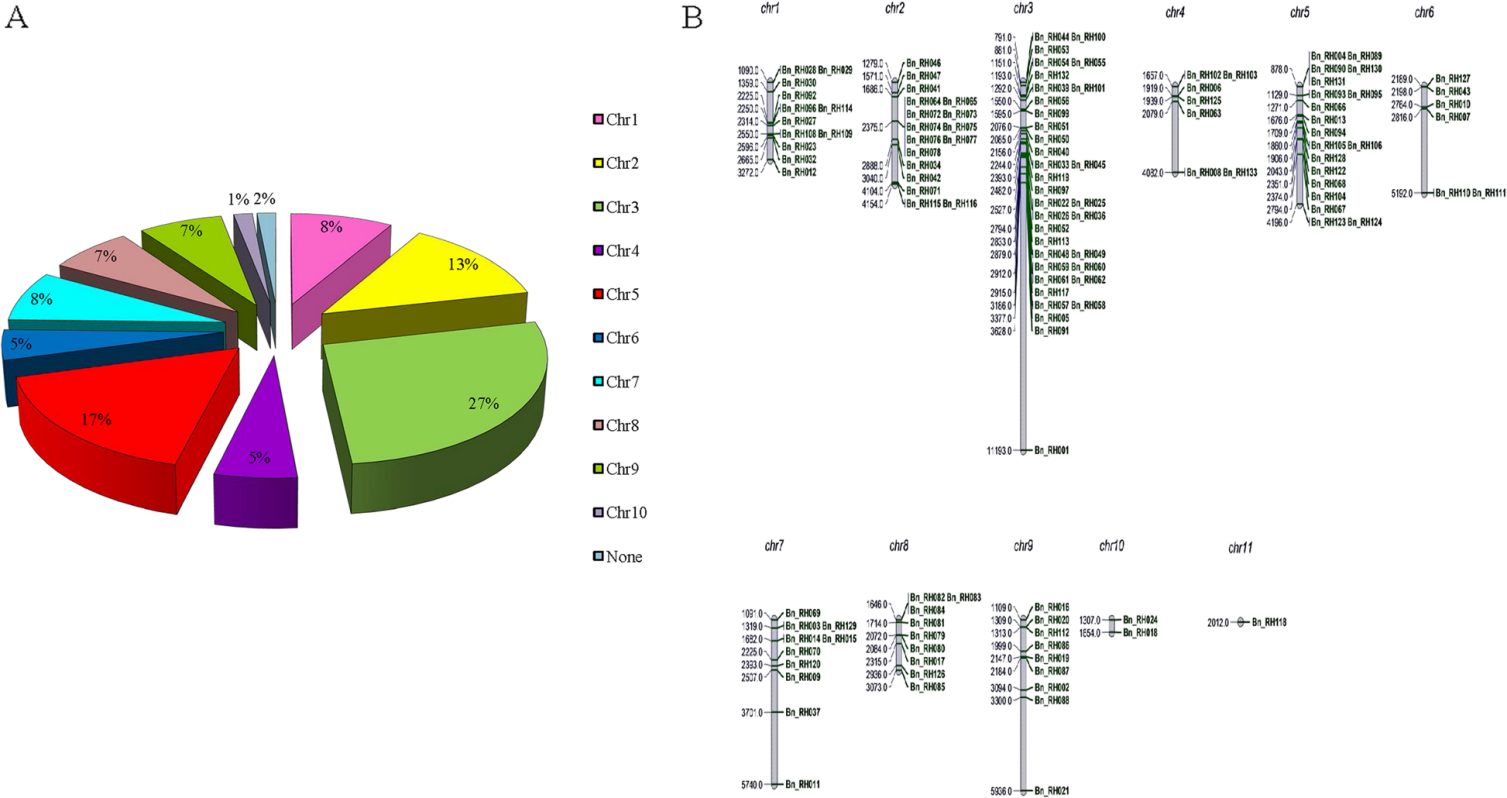
The percent of distribution frequency (**A**) and distribution (**B**) of chromosomal BnRNA-helicase proteins on rapeseed chromosomes. The numbers of chromosome are denoted above chromosome, the size of each protein in megabeses (MB) shows on the left side

 BnRHs genes are scattered and unbalanced on 10 chromosomes. Accordingly, the highest distribution is on chromosome 3 (equivalent to 27%), which indicates the importance of this chromosome, and the lowest distribution is on chromosome 10 (equivalent to 1%). Among the RHs identified, 2 genes were not assigned to any of the chromosomes (Fig. 4A and B).

### Determination of duplication and divergence of homologous genes in rapeseed

In this study, we investigated events of duplication that rise to BnRHs genes because gene duplication has an essential role in the extension of gene families and evolution. Among BnRHs, 34 genes we localized in the rejoin of tandem duplicated genes on rapeseeds chromosomes 0, 1, 2, 3, 4, 5, 6, 7, 8, and 9 (Table 3) and 25 pairs were identified as segmental duplication of genes and localized on chromosomes 0 (unknown specific location), 1, 2, 3, 4, 5, 6, 7, 8, 9, and 10 (Table 4). We found in this experimental research that’s behind the evolutionary RHs gene, the events of segmental duplication may have been the main affected force in rapeseed.

**Table 3:** Paralogous relationships between tandems duplicated pairs of RNA helicase genes in rapeseed. Chromosomal location 0 means unknown specific gene location on *B. napus* genome. Chr., Ka, Ks and λ represents chromosome, nonsynonymous, synonymous and time duplication and divergence, respectively

Gene_1	Chr.	Start1	Stop1	Start1-Stop1	Gene_2	Chr.	Start 2	Stop2	Start2-Stop2	E-Value	Ka/Ks	Ka	Ks	Ks/2ƛ
BnRH-007	6	233177	235993	2816	BnRH-030	1	1241658	1243017	1359	0	1.3118	0.2379	0.1813	13818598
BnRH-001	3	341531	342724	1193	BnRH-038	5	1247232	1248941	1709	0	1.09	0.1333	0.1223	9321646
BnRH-036	3	169756	172283	2527	BnRH-033	3	169756	172000	2244	0	0.8748	0.000875	0.000551	41967.23
BnRH-025	3	169756	172283	2527	BnRH-022	3	169756	172283	2527	0	0.8748	0.000875	0.000551	41967.23
BnRH-031	0	84287	85342	1055	BnRH-006	4	134261	136180	1919	0	1.1996	0.5231	0.4361	33239329
BnRH-027	1	168717	171031	2314	BnRH-023	1	433518	436114	2596	0	0.8217	0.002943	0.003581	272954.3
BnRH-013	5	306071	307747	1676	BnRH-009	7	1364611	1367118	2507	0	0.8486	0.1736	0.2046	15594512
BnRH-029	1	11494	12584	1090	BnRH-028	1	11494	12584	1090	0	1.3688	0.009476	0.006923	527629.6
BnRH-047	2	460676	462247	1571	BnRH-014	7	3053762	3055444	1682	0	1.3844	0.5952	0.4299	32766768
BnRH-050	3	896794	898879	2085	BnRH-048	3	896000	898879	2879	0	0.7491	0.1788	0.2387	18193598
Bn_RH011	7	28076	33816	5740	BnRH-040	3	1311916	1314072	2156	0	0.9254	0.2289	0.2473	18849085
BnRH-075	2	2633	5008	2375	BnRH-074	2	2633	5008	2375	0	0	0	1E-10	0.007622
BnRH-065	2	2633	5008	2375	BnRH-062	3	590717	593629	2912	0	0	0	1E-10	0.007622
BnRH-073	2	2633	5008	2375	BnRH-059	3	590717	593629	2912	0	1.1429	0.001558	0.001363	103874.2
BnRH-064	2	2633	5008	2375	BnRH-076	2	2633	5008	2375	0	0	0	1E-10	0.007622
BnRH-055	3	655559	656710	1151	BnRH-067	5	1522842	1525636	2794	0	0.9334	0.3113	0.3336	25426829
BnRH-068	5	1978	4329	2351	BnRH-056	3	314013	315563	1550	0	0.9634	0.3171	0.3291	25083841
BnRH-078	2	2633	5008	2375	BnRH-054	3	655559	656710	1151	0	1.0013	0.298	0.2976	22682927
BnRH-057	3	153299	156485	3186	BnRH-071	2	1036267	1040371	4104	0	0.8342	0.1178	0.1412	10762195
BnRH-079	8	240734	242806	2072	BnRH-051	3	896803	898879	2076	0	1.0233	0.043114	0.042132	3211308
BnRH-090	5	451258	452136	878	BnRH-094	5	1247232	1248941	1709	0	1.0508	0.3283	0.3124	23810976
BnRH-098	1	10280	12945	2665	BnRH-106	5	200412	202272	1860	0	0.9622	0.1644	0.1709	13025915
BnRH-109	1	43353	45903	2550	BnRH-108	1	43353	45903	2550	0	0.288	0.000399	0.001387	105708.1
BnRH-103	4	68703	70360	1657	BnRH-107	5	200412	202272	1860	0	1.0846	0.3594	0.3313	25251524
BnRH-095	5	1248996	1250125	1129	BnRH-085	8	22190	25263	3073	0	1.2089	0.2711	0.2243	17096037
BnRH-105	5	200412	202272	1860	BnRH-091	3	498725	502353	3628	0	0.9408	0.3901	0.4147	31608232
BnRH-116	2	1146345	1150499	4154	BnRH-115	2	1146345	1150499	4154	0	0.5305	0.00053	0.000323	24590.7
BnRH-114	1	472691	474941	2250	Bn_RH111	6	1333558	1338750	5192	0	0.9256	0.2283	0.2466	18795732
BnRH-126	8	239870	242806	2936	BnRH-112	9	1343959	1345272	1313	0	0.5712	0.037246	0.065207	4970027
BnRH-132	3	341531	342724	1193	BnRH-124	5	2024226	2028422	4196	0	0.9749	0.4566	0.4684	35701220
BnRH-133	4	541313	545395	4082	Bn_RH110	6	1333558	1338750	5192	0	0.9538	0.3104	0.3254	24801829
BnRH-131	5	451258	452136	878	BnRH-130	5	451258	452136	878	0	0.4357	0.000436	1E-10	0.007622
BnRH-127	6	267642	269831	2189	BnRH-120	7	1364611	1367004	2393	0	1.149	0.3859	0.3358	25594512
BnRH-121	5	11924	14339	2415	BnRH-118	5	11939	13951	2012	0	1.659	0.008056	0.004856	370086.9

**Table 4:** Paralogous relationships between segmental pairs of RNA helicase genes in rapeseed. Chromosomal location 0 means unknown specific gene location on *B. napus* genome. Chr., Ka, Ks and λ represents chromosome, nonsynonymous, synonymous and time duplication and divergence, respectively

Gene_1	Chr.	Start1	Stop1	Start2-Stop2	Gene_2	Chr.	Start 2	Stop2	Start2-Stop2	Ka/Ks	Ka	Ks	E-Value	Ks/2ƛ
BnRH-020	9	155278	156587	1309	BnRH-035	0	108823	111283	2460	0.5034	0.02242	0.044545	0	3395160.1
BnRH-019	9	374305	376452	2147	BnRH-024	10	69125	70432	1307	1.0232	0.3585	0.3504	0	26707317
BnRH-043	6	267642	269840	2198	BnRH-018	10	19064	20718	1654	0.9503	0.3277	0.3449	0	26288110
BnRH-004	5	451258	452136	878	BnRH-012	1	146054	149326	3272	0.9206	0.3519	0.3822	0	29131098
BnRH-005	3	1494980	1498357	3377	BnRH-003	7	209724	211043	1319	1.0456	0.4534	0.4336	0	33048780
BnRH-021	9	295276	301212	5936	BnRH-032	1	10280	12945	2665	0.9793	0.8722	0.8906	0	67881098
BnRH-037	7	3665918	3669619	3701	BnRH-034	2	491779	494667	2888	1.0309	0.2948	0.2859	0	21791159
BnRH-044	3	1134199	1134990	791	BnRH-049	3	896000	898879	2879	1.0882	0.2365	0.2173	0	16562500
BnRH-008	4	541313	545395	4082	BnRH-010	6	420376	423140	2764	1.0468	0.4405	0.4208	0	32073171
BnRH-041	2	591701	593387	1686	BnRH-002	9	208559	211653	3094	0.8414	0.04603	0.054706	0	4169643.3
BnRH-060	3	590717	593629	2912	BnRH-072	2	2633	5008	2375	0.9149	0.07868	0.085991	0	6554211.1
BnRH-061	3	590717	593629	2912	BnRH-077	2	2633	5008	2375	0.9473	0.07912	0.083519	0	6365742.4
BnRH-069	7	3053762	3054853	1091	BnRH-052	3	229605	232399	2794	0.907	0.2966	0.327	0	24923780
BnRH-066	5	1524365	1525636	1271	BnRH-058	3	153299	156485	3186	1.2125	0.3236	0.2669	0	20342988
BnRH-053	3	1138324	1139205	881	BnRH-050	3	896794	898879	2085	1.0637	0.2186	0.2055	0	15663110
BnRH-101	3	677191	678483	1292	BnRH-097	3	524321	526803	2482	1.0123	0.2922	0.2886	0	21996951
BnRH-082	8	462969	464615	1646	BnRH-102	4	68703	70360	1657	1.3324	0.3288	0.2467	0	18803354
BnRH-104	5	1747505	1749879	2374	BnRH-089	5	451258	452136	878	0.9073	0.5462	0.6021	0	45891768
BnRH-099	3	1103675	1105270	1595	BnRH-081	8	241092	242806	1714	0.8312	0.03467	0.04171	0	3179142.5
BnRH-096	1	472691	474941	2250	BnRH-092	1	94903	97128	2225	1.1518	0.2171	0.1885	0	14367378
BnRH-043	6	267642	269840	2198	BnRH-018	10	19064	20718	1654	0.9503	0.3277	0.3449	0	26288110
BnRH-100	3	1134199	1134990	791	BnRH-080	8	240722	242806	2084	1.0791	0.2371	0.2197	0	16745427
BnRH-087	9	1343959	1346143	2184	BnRH-083	8	462969	464615	1646	1.1999	0.2959	0.2466	0	18795732
BnRH-117	3	590714	593629	2915	BnRH-129	7	209724	211043	1319	1.222	0.5148	0.4213	0	32111280
BnRH-100	3	1134199	1134990	791	BnRH-080	8	240722	242806	2084	1.0791	0.2371	0.2197	0	16745427

We are analysis of synteny and gene duplication of BnRHs among *Arabidopsis* and *Solanum lycopersocum* through 19 chromosomes of *Brassica napus*. In order to recognize the evolutionary origin of BnRHs, the syntenic comparative analysis was conducted among rapeseed and *Arabidopsis*, and tomato. We estimated synonymous (Ks) and nonsynonymous (Ka) substitution rates (Ka/Ks) for the evaluation of positive selection pressure after duplication. The Ka/Ks ratio for tandem duplication genes ranged from 0 to 1.659 with an average of 0.885, while Ka/Ks for the segmental duplication genes was 0.5034 to 1.3324 with a mean of 1.009. In addition, the Ka/Ks ratio of orthologous gene pairs between rapeseed and other two plant species was calculated (Additional files 2 and 3). The mean Ka/Ks was between rapeseed and *Arabidopsis* (0.7983), rapeseed and tomato (0.7549), respectively, deciphering that the genetic pairs between *Brassica napus* and the other two species are strongly subjected to pure selection.

### Analysis of promoter regions of BnRHs genes in rapeseed

*Cis-*regulatory elements play an essential role in determining the properties of different tissues, especially under stress conditions. To evaluate the function of BnRHs genes in rapeseed, upstream to 2000 bp of each gene were examined for stress response. Therefore, the promoter region of BnRHs gene sequences was identified using the PLACE database in terms of the type of regulatory elements and their role. For example, elements S000133, S000173, and S000415 for drought stress, S000418 for salinity stress, S000407 for cold stress, and S000030 were identified for heat stress. The results indicate that BnRHs genes are responding to abiotic stresses and increasing plant resistance to these stresses. The highest number of elements respectively in drought stress related to element S000415 with ACGTG sequence (460), in salinity stress related to element S000453 with sequence GAAAAA (1190), and in heat stress related to element S000030 with sequence CCAAT (341) play an essential role in abiotic-stress response (Additional file 4).

### Physiological and relative expression of BnRHs genes during abiotic stresses

The role of BnRHs genes in responding to environmental stress conditions, and gene expression analyses were performed using qRT-PCR in rapeseed plants subjected to drought, salt, heat, cold, and cadmium treatments. Furthermore, the effect of these stress conditions on the physiological and molecular characteristics of plants was evaluated by measuring changes in RWC, sodium, potassium, and proline concentration in both leaf and root tissues of Hayolla#50 and #4815 cultivars.

**Fig. 5:**
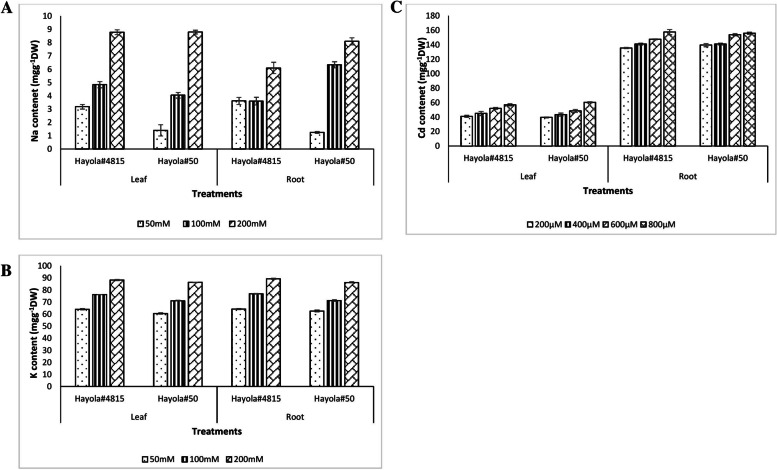
Na (**A**), K (**B**) and Cd content (**C**) in leaf and root tissues of Hayola #50 and Hayola #4815 rapeseed cultivars in response to salt and cadmium stress

**Fig. 6:**
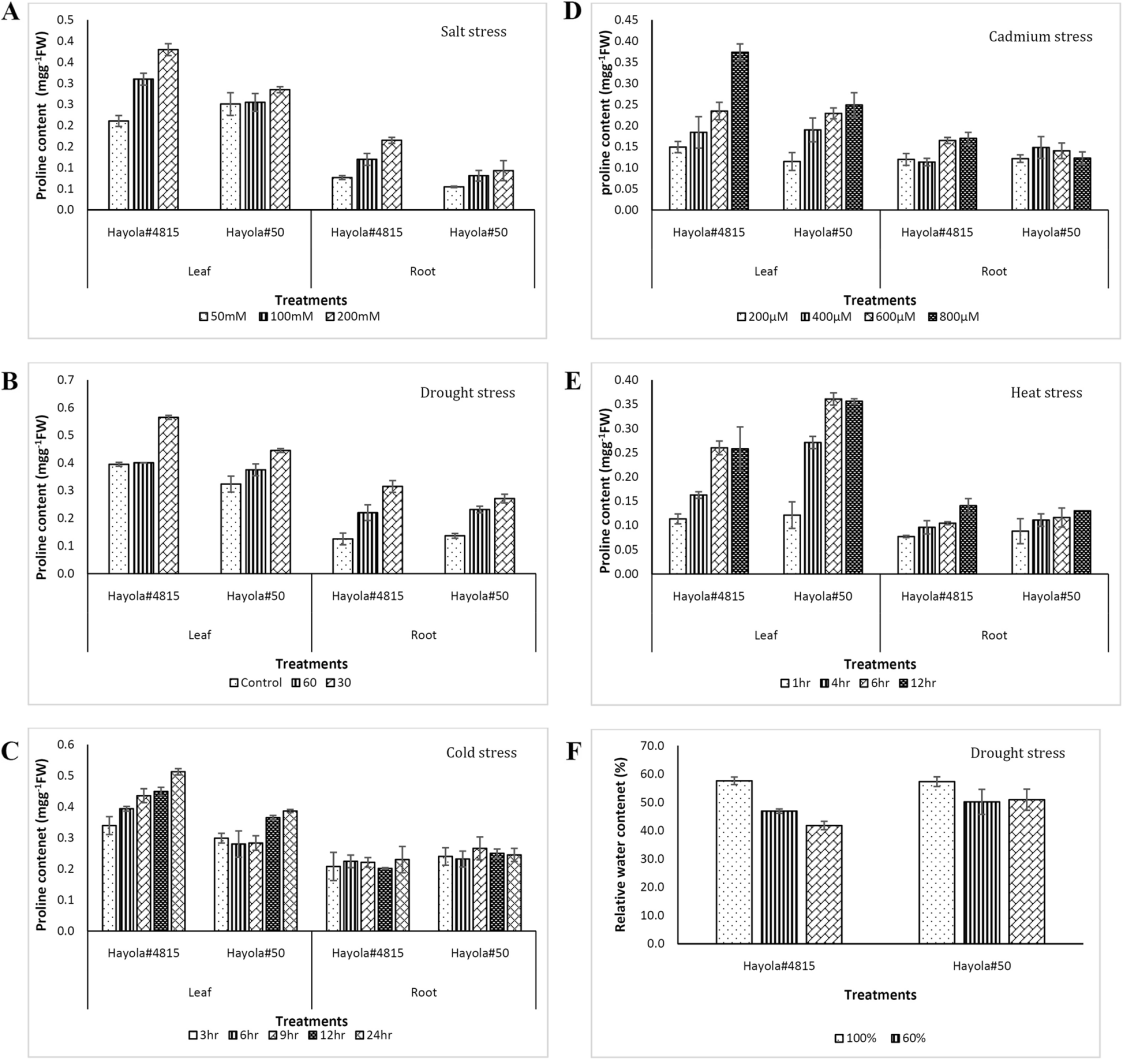
Proline content (**A**-**E**) in leaf and root tissues and relative water content (**F**) of Hayola #50 and Hayola #4815 rapeseed cultivars in response to salt, drought, cold, and cadmium stresses

 The results obtained from RWC analyses demonstrated that the RWC in leaf tissue decreased with higher levels of drought stress (Additional file 5). This decrease led to growth deficiency and some physiological and metabolic alterations in two rapeseed cultivars. According to the results of sodium and potassium concentration analyses, it was shown that the sodium concentration in both root and leaf tissues was increased upon increased levels of stress treatments. This increase in Na^+^ contents of plant tissues led to an increase in osmotic potential and a decrease in K^+^ uptake (Figs. 5 and 6). The significant effects of high Na^+^ accumulation in root, transport of Na^+^ has been limited to leaves, so a decrease in K^+^/Na^+^ ratio was observed in the treated plants compared to control plants (Additional file 6). The proline content of both root and leaf tissues was evaluated and was found to be increased in both leaf and root tissues with the increased level of stress treatment. This increase was considered an indicator of plant resistance to related stress conditions. The results confirmed the effects of various stress conditions on the two rapeseed cultivars studied and showed that the plants were affected by stress exposure (Additional files 7 and 8).

Hayola#50 and #4815 rapeseed genotypes have significant differences in terms of Na^+^ and K^+^ levels in leaf and root tissues at *p* < 0.01, and this shows the different tolerance of the two genotypes at different levels of salinity.

### Expression pattern of BnRHs genes in response to drought stress

**Fig. 7:**
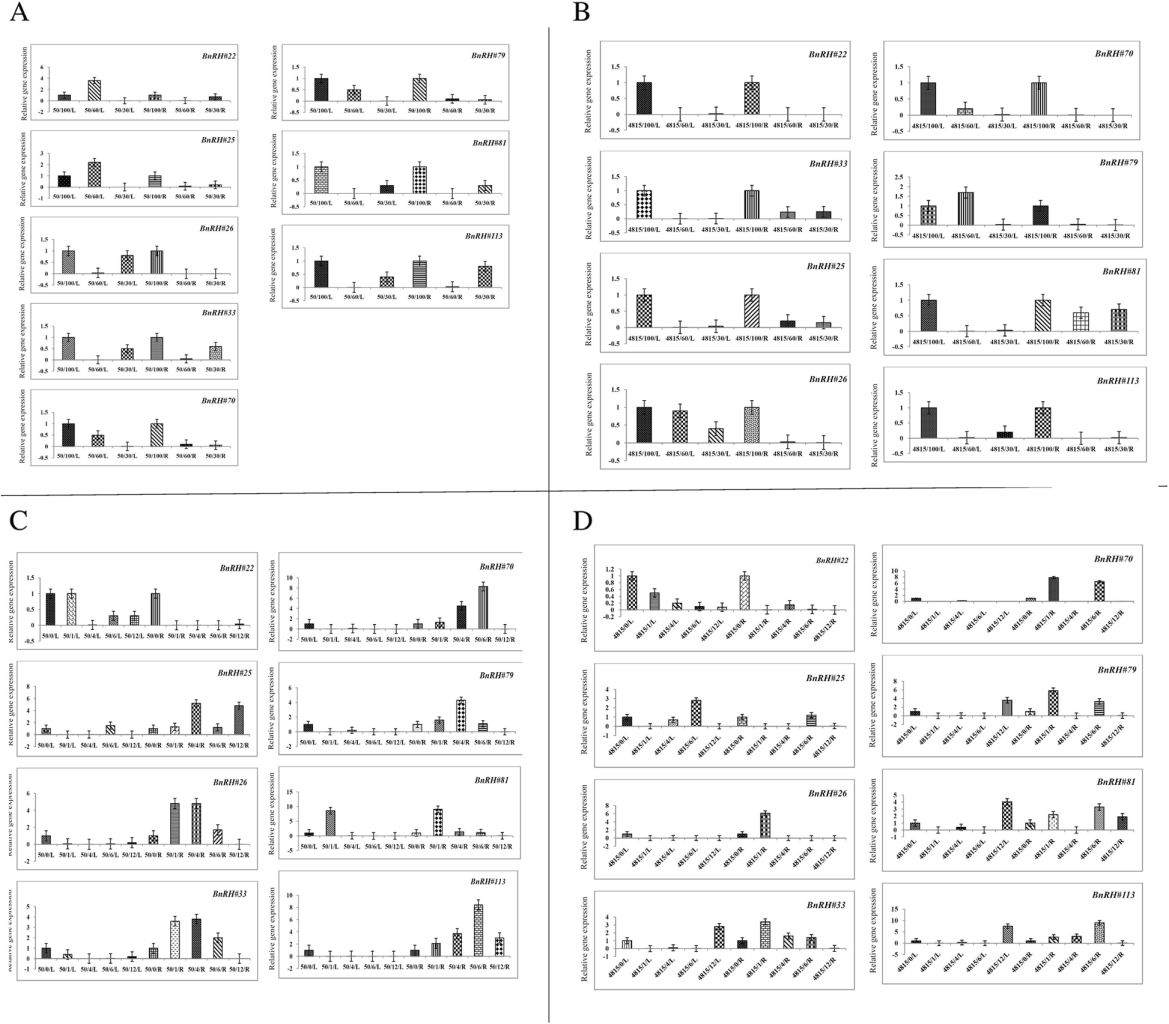
Relative gene expression of studied BnRH genes in two rapeseed cultivars Hayola #50 and #4815 in response to drought (**A**, **B**) and heat (**C**, **D**) stress in leaf and root tissues. 50, 4815, L and R represented Hayola #50 and #4815, leaf and root tissues of rapeseed cultivars. Drought stress were subjected to three optimal irrigation regimes (field capacity (planting capacity, FC = 100%), 30 and 60% and heat stress levels including 25 ± 1 °C (control) and 37 ± 1 °C (heat stress) at interval 1, 4, 6, and 12 h. The name of each gene was denoted in the above side

 Expression pattern of BnRHs genes in response to drought stress of Hayola#4815, the results showed that all BnRHs genes except *BnRH#79* at 60% drought stress with 1.7 fold changes up-regulated, in both leaves and roots had less expression than the control level and were negatively regulated (Fig. 7A; Table 5). In addition, the expression pattern of BnRHs genes in response to drought stress of Hayola#50, showed gene expression in *BnRH#22* and *BnRH#25* was at 60% of high leaf capacity with 3.6 and 2.2 fold changes up-regulated, and in other BnRHs genes, relative gene expression in both leaves and roots was equal and less than the control level which down-regulated (Fig. 7B; Table 6).

**Table 5:** Expression of BnRH genes response to abiotic stress in *Brassica napus* L. var. Hayola #4815

BnRH gene name	Drought stress						Salt stress								Heat stress									
Hayolla #4815																								
**Tissue**	**leaf**			**Root**			**leaf**				**Root**				**Leaf**					**Root**				
	**100**	**60**	**30**	**100**	**60**	**30**	**0**	**50**	**100**	**200**	**0**	**50**	**100**	**200**	**0**	**1**	**4**	**6**	**12**	**0**	**1**	**4**	**6**	**12**
*BnRH#22*	1	0↓	0.02↓	1	0↓	0↓	1	0.4↓	0.03↓	0.6↓	1	0.6↓	0.01↓	0.12↓	1	0.5↓	0.2↓	0.1	0.08↓	1	0.006↓	0.15↓	0.02	0.0007↓
*BnRH#25*	1	0.005↓	0.004↓	1	0.2↓	0.15↓	1	0.06↓	0.0003↓	0.0005↓	1	0.6↓	0.004↓	0.5↓	1	0.009↓	0.7↓	2.8↑	0.002↓	1	0.0009↓	0.0006↓	1.2↑	0.03↓
*BnRH#26*	1	0.9↓	0.4↓	1	0.03↓	0.009↓	1	9.0↑	0.02↓	0.0002↓	1	1.1↓	0.0004↓	4.4↑	1	0.0007↓	0.06↓	0.02↓	0.0004↓	1	6.1↑	0.0005↓	0.0003	0.004↓
*BnRH#33*	1	0.0006↓	0.009↓	1	0.24↓	0.25↓	1	5.1↑	0.1↓	1.7↑	1	0.0002↓	0.02↓	3.8↑	1	0.0002↓	0.11↓	0.009↓	2.8↑	1	3.4↑	1.6↑	1.4↑	0.03↓
*BnRH#70*	1	0.02↓	0.02↓	1	0.009↓	0.0002↓	1	5.3↑	3.2↑	4.8↑	1	4.3↑	0.0006↓	8.5↑	1	0.0003↓	0.2↓	0.0008↓	0.002↓	1	7.8↑	0.0004↓	6.5↑	0.02↓
*BnRH#79*	1	1.7↑	0.03↓	1	0.04↓	0.008↓	1	6.99↑	0.05↓	0.001↓	1	5.2↑	0.0009↓	0.0004↓	1	0.0004↓	0.06↓	0.0004↓	3.6↑	1	5.8↑	0.0004↓	3.3↑	0.05↓
*BnRH#81*	1	0.006↓	0.03↓	1	0.6↓	0.7↓	1	0.001↓	0.05↓	0.001↓	1	0.001↓	0.008↓	2.9↑	1	0.002↓	0.4↓	0.003↓	4.04↑	1	2.2↑	0.002↓	3.3↑	1.9↑
*BnRH#113*	1	0.02↓	0.2↓	1	0.001↓	0.02↓	1	0.2↓	3.0↑	0.01↓	1	0.006↓	0.0002↓	0.2↓	1	0.0002↓	0.3↓	0.0007↓	7.5↑	1	2.7↑	3.0↑	9.0↑	0.03↓
		ns	ns		ns	ns		*	ns	ns		ns	ns	*		ns	*	ns	ns		*	ns	*	ns
	**Cold stress**												**Cadmium stress**											
**Tissue**	**leaf**						**Root**						**Leaf**					**Root**						
	**0**	**3**	**6**	**9**	**12**	**24**	**0**	**3**	**6**	**9**	**12**	**24**	**0**	**200**	**400**	**600**	**800**	**0**	**200**	**400**	**600**	**800**		
*BnRH#22*	1	0.11↓	1.5↑	4.7↑	9.3↑	8.3↑	1	1.31↑	1.4	4.3↑	3.2↑	1.1↓	1	0.16↓	0.003↓	0.03↓	0.04↓	1	0.001↓	0.4↓	0.06↓	0.003↓		
*BnRH#25*	1	4.7↑	9.0↑	0.7↓	5.1↑	2.1↑	1	3.8↑	6.9↑	5.2↑	0.05↓	5.1↑	1	3.8↑	0.0003↓	7.9↑	0.08↓	1	2.6↑	9.8↑	0.07↓	0.3↓		
*BnRH#26*	1	2.3↑	0.004	0.0002↓	3.8↑	0.0002	1	7.3↑	3.0↑	5.0↑	0.0002↓	4.8↑	1	0.0003↓	3.3↑	1.14↑	0.6↓	1	1.8↑	6.8↑	0.6↓	0.006↓		
*BnRH#33*	1	7.3↑	2.1↑	0.002↓	3.3↑	5.7↑	1	3.8↑	7.3↑	1.8↑	0.0002↓	1.3↓	1	3.99↑	0.002↓	0.02↓	1.05↓	1	2.05↑	2.09↑	1.8↑	2.5↑		
*BnRH#70*	1	2.0↑	2.0↑	1.6↑	1.5↑	6.0↑	1	7.4↑	6.5↑	1.8↑	0.03↓	2.6↑	1	0.26↓	4.3↑	1.5↑	0.8↓	1	0.008↓	1.6↑	0.4↓	0.2↓		
*BnRH#79*	1	0.002↓	0.0004	3.0↑	0.02↓	1.5↑	1	1.9↑	1.5↑	1.1↓	1.1↓	3.5↑	1	2.4↑	0.011↓	1.9↑	0.05↓	1	2.4↑	1.3↑	0.8↓	0.03↓		
*BnRH#81*	1	7.0↑	6.0↑	5.4↑	1.1↓	4.0↑	1	4.2↑	1.2↓	6.4↑	2.5↑	2.0↑	1	3.0↑	0.0002↓	0.0008↓	1.7↑	1	1.5↑	5.2↑	5.2↑	2.5↑		
*BnRH#113*	1	0.05↓	0.002↓	0.4↓	0.02↓	0.04	1	7.0↑	0.3↓	0.8	0.008↓	0.02	1	1.0↓	0.05↓	0.004↓	0.2↓	1	0.03↓	0.4↓	0.3↓	0.004↓		
		*	ns	*	*	*		**	*	**	ns	*		*	ns	ns	*		*	*	ns	ns		

Rapeseed seedling with fully expanded leaves were treated by abiotic stress including drought for three optimal irrigation regimes (field capacity, FC = 100%), 30 and 60%, saline treatments including 50, 100, and 200 mM NaCl, heat stress were subjected to 25 ± 1 °C (control) and 37 ± 1 °C (heat stress) and then at intervals of 1, 4, 6, and 12 h

Cold stress at 4 °C in the cold room, and then at intervals of 3, 6, 9, 12, and 24 h and cadmium stress including 0 (control), 200, 400, 600, and 800 µM cadmium chloride

Number shows the fold change in relative to control

↑the expression is up-regulated

↓the expression is down-regulated

*, ** and ns represent the test significant at *p* < 0.05, *p* < 0.01 and not significant, respectively

**Table 6:** Expression of BnRH genes response to abiotic stress in Brassica napus L. var. Hayola #50

BnRH gene name	Drought stress						Salt stress								Heat stress									
Cultivar Hayola #50																								
**Tissue**	**leaf**			**Root**			**leaf**				**Root**				**Leaf**					**Root**				
	**100**	**60**	**30**	**100**	**60**	**30**	**0**	**50**	**100**	**200**	**0**	**50**	**100**	**200**	**0**	**1**	**4**	**6**	**12**	**0**	**1**	**4**	**6**	**12**
*BnRH#22*	1	3.6↑	0.004↓	1	0.02↓	0.7↓	1	0.0007↓	0.1↓	0.2↓	1	0.002↓	0.04↓	4.3↑	1	1.0	0.01↓	0.3	0.3↓	1	0.0003↓	0.0005↓	0.0004↓	0.04↓
*BnRH#25*	1	2.2↑	0↓	1	0.08↓	0.2↓	1	0.002↓	0.006↓	0.0002↓	1	0.004↓	0.0004↓	7.7↑	1	0.002↓	0.02↓	1.5↑	0.007↓	1	1.3↑	5.2↑	1.2↑	4.8↑
*BnRH#26*	1	0.04↓	0.8↓	1	0.0006↓	0.005↓	1	1.2↓	0.0002↓	5.7↑	1	6.2↑	7.0↑	8.0↑	1	0.06↓	0.0009↓	0.05	0.2↓	1	4.8↑	4.8↑	1.7↑	0.0002↓
*BnRH#33*	1	0.004↓	0.5↓	1	0.05↓	0.6↓	1	3.7↑	4.6↑	4.0↑	1	3.9↑	2.8↑	3.9↑	1	0.4↓	0.002↓	0.02↓	0.2↓	1	3.6↑	3.8↑	2.0↑	0.002↓
*BnRH#70*	1	0.5↓	0.005↓	1	0.1↓	0.06↓	1	0.0002↓	0.003↓	0.0007↓	1	0.0002↓	0.0008↓	1.4↑	1	0.004↓	0.09↓	0.0008↓	0.0004↓	1	1.3↑	4.5↑	8.3↑	0.00002↓
*BnRH#79*	1	0.002↓	0	1	0.1↓	0.4↓	1	8.0↑	0.003↓	0.05↓	1	0.007↓	0.02↓	1.2↑	1	0.002↓	0.2↓	0.0004↓	0.0006↓	1	1.6↑	4.3↑	1.1↓	0.0003↓
*BnRH#81*	1	0	0.3↓	1	0	0.3↓	1	0.0003↓	0.004↓	0.001↓	1	0.02↓	0.003↓	6.6↑	1	8.6↑	0.06↓	0.003↓	0.004↓	1	9.1↑	1.4↑	1.1↓	0.04↓
*BnRH#113*	1	0.003↓	0.4↓	1	0.03↓	0.8↓	1	0.02↓	0.004↓	0.2↓	1	0.0004↓	0.0004↓	0.5	1	0.0002↓	0.02↓	0.002↓	0.008↓	1	2.1↑	3.7↑	8.4↑	3.0↑
		ns	ns		*	**		ns	ns	ns		ns	ns	**		ns	ns	ns	ns		*	**	*	ns
	**Cold stress**												**Cadmium stress**											
**Tissue**	**Leaf**						**Root**						**Leaf**					**Root**						
	**0**	**3**	**6**	**9**	**12**	**24**	**0**	**3**	**6**	**9**	**12**	**24**	**0**	**200**	**400**	**600**	**800**	**0**	**200**	**400**	**600**	**800**		
*BnRH#22*	1	0.01↓	4.0↑	0.002↓	0.003↓	0.0005↓	1	1.03↑	0.03↓	3.0↑	1.6↑	5.0↑	1	0.7↓	0.16↓	0.72↓	0.006↓	1	0.0003↓	0.03↓	0.013↓	0.001↓		
*BnRH#25*	1	0.02↓	1.6↑	8.1↑	0.007↓	0.05↓	1	0.001↓	1.6↑	7.2↑	3.08↑	5.3↑	1	0.13↓	0.04↓	0.07↓	0.00015↓	1	9.0↑	0.1↓	0.11↓	2.4↑		
*BnRH#26*	1	0.06↓	0.0005↓	0.0006↓	0.005↓	0.0006↓	1	3.8↑	3.81↑	4.9↑	1.04↓	3.0↑	1	0.3↓	0.3↓	0.45↓	0.04↓	1	1.4↑	0.02↓	0.02↓	9.9↑		
*BnRH#33*	1	0.007↓	0.002↓	0.0009↓	0.09↓	0.0008↓	1	5.6↑	2.6↑	2.04↑	3.1↑	1.2↓	1	2.6↑	2.4↑	8.1↑	0.02↓	1	6.2↑	1.01	1.5↑	3.9↑		
*BnRH#70*	1	0.0003↓	0.002↓	0.0005↓	0.0003↓	2.1↑	1	1.5↑	8.2↑	6.5↑	3.2↑	0.4↓	1	0.08↓	0.03	0.02↓	0.002↓	1	8.02↑	0.2↓	0.4↓	0.2↓		
*BnRH#79*	1	0.004↓	3.1↑	6.0↑	0.04↓	1.8↑	1	2.3↑	1.6↑	2.1↑	1.1↓	4.5↑	1	0.002↓	1.7↑	0.07↓	0.0003↓	1	0.0099↓	2.3↑	0.07↓	0.7↓		
*BnRH#81*	1	0.02↓	3.3↑	0.0005↓	0.007↓	0.0003↓	1	4.2↑	1.5↑	3.2↑	1.6↑	1.2↓	1	1.7↑	1.5↑	3.0↑	0.002↓	1	3.4↑	1.5↑	1.1↓	0.0002↓		
*BnRH#113*	1	4.4↑	0.006↓	0.005↓	0.002↓	0.002↓	1	0.009↓	0.3↓	1.03	1.6↑	1.0↓	1	0.06↓	0.08↓	0.4↓	0.003↓	1	0.05↓	0.06↓	0.03↓	0.004↓		
		ns	*	ns	ns	ns		*	*	**	***	**		ns	ns	ns	ns		*	ns	ns	ns		

Rapeseed seedling with fully expanded leaves were treated by abiotic stress including drought for three optimal irrigation regimes (field capacity, FC = 100%), 30 and 60%, saline treatments including 50, 100, and 200 mM NaCl, heat stress were subjected to 25± 1°C (control) and 37 ± 1°C (heat stress) and then at intervals of 1, 4, 6, and 12 hours

Cold stress at 4 °C in the cold room, and then at intervals of 3, 6, 9, 12, and 24 h and cadmium stress including 0 (control), 200, 400, 600, and 800 μM cadmium chloride

Number shows the fold change in relative to control

↑the expression is up-regulated

↓the expression is down-regulated

*, ** and ns represent the test significant at *p*<0.05, *p*<0.01 and not significant, respectively

### Expression pattern of BnRHs genes in response to heat stress

In Hayola#4815 cultivar, the result showed that in *BnRH#22*, all samples had less expression than the control level. In other BnRHs genes, including *BnRH#25*, 6-hour heat level of leaves and roots with 2.8 fold change increased expression. In *BnRH#26*, all samples except a one-hour heat level in roots with 6.1 fold change other level of treatments have been down-regulated; in *BnRH#33*, 12-hour level in leaves with 2.8 fold change and 1, 4 and 6 h of heat stress at the roots with 3.4, 1.6 and 1.4 fold changes, respectively had an expressive expression and up-regulated (Fig. 7C; Table 5). The results of expression of the studied BnRHs genes in response to heat stress of Hayola#50 showed that *BnRH#22* had lower expression than control for all samples and down-regulated. In other BnRHs genes for *BnRH#25*, levels of 6 h per leaf with 1.5 fold change and in all root levels, respectively, in *BnRH#26*, #33, #70, and #79, level one, four and 6 h of the root significantly up-regulated, in *BnRH#81*, level of one hour of root with 9.1 fold change at *p* < 0.05 significantly up-regulated and leaf with 8.6 fold change and high expression, in *BnRH#113*, all leaf samples at all treatment levels had lower expression non-significantly regulated and all samples of root at treatments had significantly increased expression than control conditions (Fig. 7D; Table 6).

### Expression pattern of BnRHs genes in response to cold stress

**Fig. 8:**
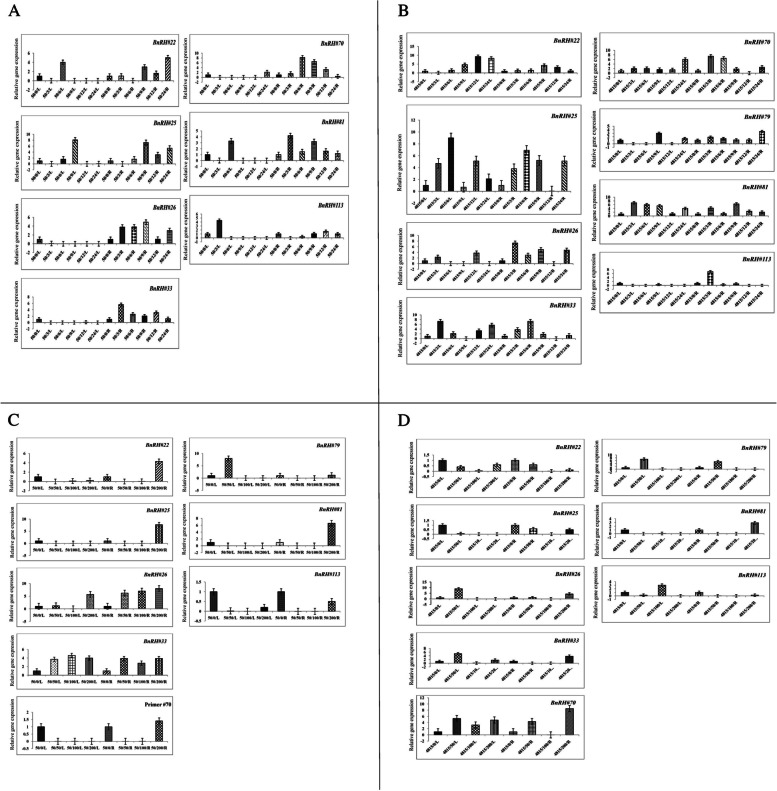
Relative gene expression of studied BnRH genes in two rapeseed cultivars Hayola #50 and #4815 in response to cold (**A**, **B**) and salt (**C**, **D**) stress. 50, 4815, L and R represented Hayola #50 and #4815, leaf and root tissues of rapeseed cultivars. The cold stress at 4 °C in the cold room, at intervals of 3, 6, 9, 12, and 24 h, was subjected. Saline treatments were including 50, 100, and 200 mM NaCl

 In Hayola#4815 cultivar, all of the samples leaves and roots had increased expression compared to the control, and it can be concluded that rapeseed is a cold-loving plant (Table 5). The expression pattern of BnRHs genes in response to cold stress in Hayola#50 increased at the level of 6 h of leaves and increased expression at the levels of 9, 12, and 24 h, respectively. In *BnRH#70*, all samples had increased expression except at the 24 h level at the root with maximum fold change (8.2) at *p* < 0.05 significantly up-regulated and decreased expression at leaf level. In *BnRH#79*, all leaf samples except level 3 h had increased expression and in root at 24 h with 4.5 fold change significantly (*p* < 0.01) up-regulated had more expression than control at all levels. In *BnRH#113*, all samples except the three-hour treatment level with 4.4 fold change, had lower expression, and 12 h levels (1.6 fold change) had increased expression at the root (Fig. 8 A, B, Table 6).

### Expression pattern of BnRHs genes in response to salinity stress

Expression pattern of BnRHs genes in response to salinity stress in Hayola#4815 cultivar showed that in *BRH#22*, #25, and #26 with reduced expression, in *BnRH#33* except 200 mM level in the root with 3.8 fold change, all have less expression and down-regulated, in *BnRH#70*, except 100 mM root level, all samples have high expression, which at 200 mM level with 8.5 fold change at *p* < 0.01 significantly up-regulated. *BnRH#81* except 200 mM root level (2.9 fold change) of all samples had low expression and in *BnRH#113*, except 100 mM (with 3 fold change), all samples of leaf had less expression than the control (Fig. 8C; Table 5). Hyola#50 cultivar in response to salinity stress in *BnRH#22* and #25, respectively, except the level of 200 mM of the root (4.3 and 7.7. fold change) all have less expression than the control, in *BnRH#26* all samples at the root and the level of 200 mM in the leaf with increased expression, in *BnRH#33* all samples in leaves and roots have high increase, in *BnRH#70* all samples in leaves and roots except 200 mM level with 1.4 fold change have decreased expression, in *BnRH#79* all samples except 50 mM level (with 8 fold change) in leaves with reduced expression, in *BnRH#81*, except for the level of 200 mM with 6.6 fold change with significantly up-regulation, the root had low expression and in *BnRH#113*, all leaf and root samples had less expression than the control (Fig. 8D; Table 6).

### Expression pattern of BnRHs genes in response to cadmium stress

**Fig. 9:**
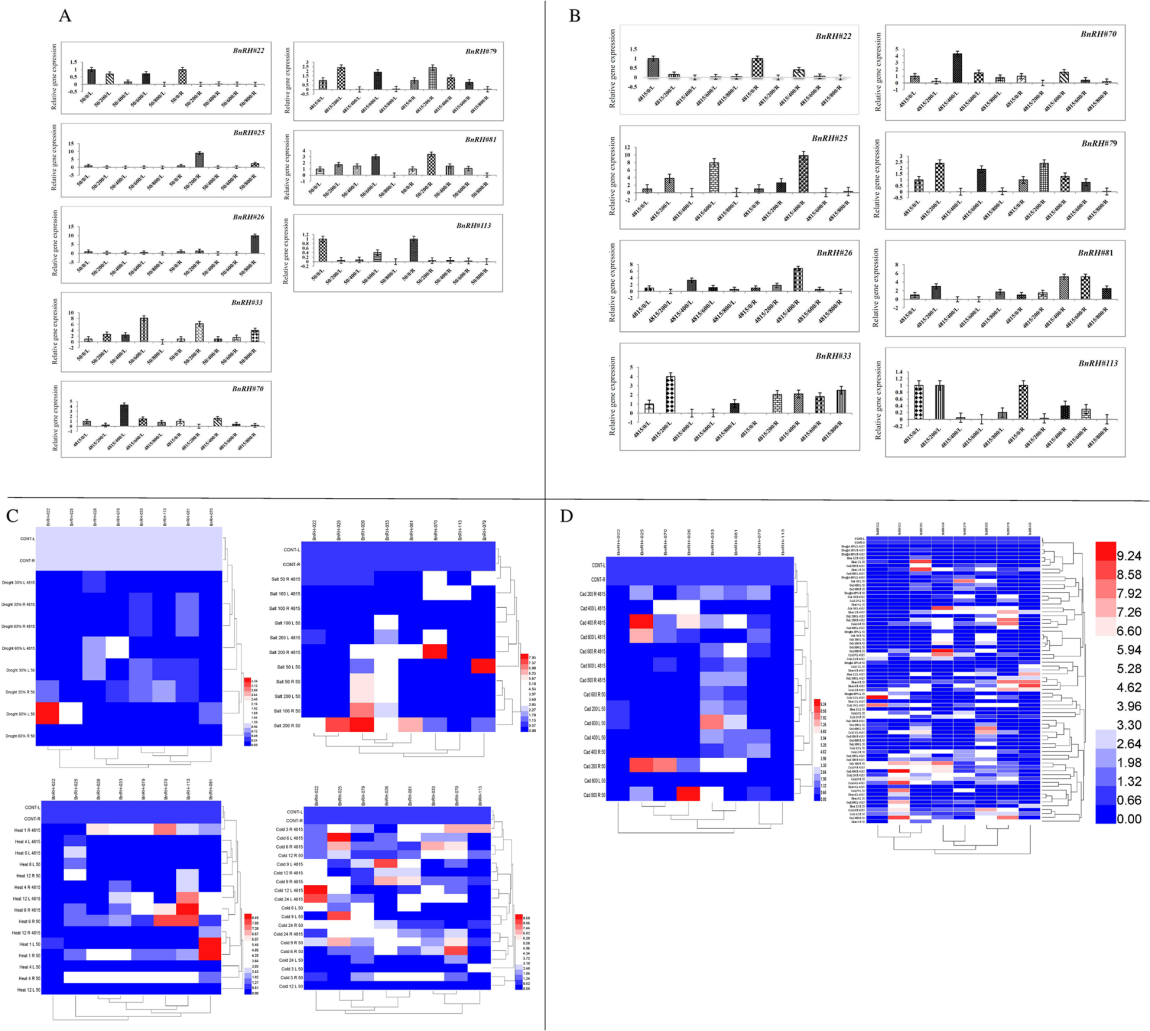
Relative gene expression of studied BnRH genes in two rapeseed cultivars Hayola #50 and #4815 in response to cadmium stress (**A**, **B**) and heat map of studied genes in response to drought, heat, cold, salt, cadmium and total of stress (**C**, **D**). 50, 4815, L and R represented Hayola #50 and #4815, leaf and root tissues of rapeseed cultivars. The cadmium concentrations of 0 (control), 200, 400, 600, and 800 µM cadmium chloride was subjected

 Expression pattern of BnRHs genes in response to cadmium stress in Hayola#4815 has the lowest expression in *BnRH#22*, *#25* and *#26*; in *BnRH#33*, all samples have the highest expression in root and 200 µM level in leaf, in *BnRH#70* except level 400 (with 4.3 fold change) and 600 µM (with 1.5 fold change) in leaf and 400 µM in root, all samples with the lowest expression, in *BnRH#79* except level 800 µM at root and leaf and level 400 µM leaf, all samples have the highest rate of expression, in *BnRH#81* all samples at root and level 200 µM with 3.0 fold change significantly up-regulated in leaf increased expression. Finally, all leaf and root samples in *BnRH#113* at all levels of treatment had a relative decrease in expression compared to the control (Fig. 9A; Table 5). Hayola #50 in response to cadmium stress in *BnRH#22*, *#25*, and *#26* had the lowest expression, in *BnRH#33* except 800 µM (8.1 fold change) in leaf all samples had high expression, in *BnRH#70* all samples except 200 µM (8.02 fold change) in root had low expression, In *BnRH#79*, except 400 µM (2.3 fold change) in root level and 400 µM (1.7 fold change) leaf level, there was a decrease in expression. In *BnRH#81*, except for 800 µM leaf and root, all samples had high expression, and in *BnRH#113*, all samples had reduced expression compared to control (Fig. 9B; Table 6).

### Heat map of BnRHs genes under the influence of abiotic stresses

Heat maps are a powerful tool for visualizing gene expression data in order to allow researchers to quickly and easily identify patterns of gene expression that are associated with specific conditions or treatment. The results of the heat map of the expression pattern of RHs genes in leaf and root tissues of selected genes separately show that the studied genes in terms of expression pattern under cadmium, cold, drought, salinity, and heat stress (Fig. 9C). They are divided into four and five separate groups, respectively. The heat map of the composition of the studied genes in terms of expression profile for all abiotic stresses has divided them into four groups (Fig. 9D).

## Discussion

Environmental stresses are known to affect cellular gene expression and crop production [6]. Therefore, in the face of abiotic stresses plants have shown various regulatory mechanisms in response to abiotic stresses that cause tolerance to such environmental conditions [4]. Molecular breeding has extensively employed with the main goal of developing abiotic stress tolerant rapeseed varieties. Recent progress in high-throughput technologies to develop abiotic stress tolerance rapeseed. Gene documentation and investigation of trait in rapeseed using genomic tools has expanded the abilities for molecular breeding combined with up-to-date tools of genetic improvement [1]. So, helicases are the molecules to be affected in response to stress and represent a large protein family that is classified in RNA metabolism, which is illustrated in the domain of eukaryotes and prokaryotes [43]. These enzymes play a significant role in gene regulation and expression. RHs are involved in the modification and synthesis of ribonucleotides, RNPs, and pre-mRNA binding. The availability of genome sequences has enabled deciphering this family of genes in various plant species, including *Arabidopsis*, rice, tomato, maize, and soybean [44–47]. However, in rapeseed no RHs have been characterized. Therefore, the majority of biological functions of RHs require further investigation.

In this study, we presented the complete survey of the RH gene family and the possible role of RHs genes mechanism in rapeseed in response to abiotic stress under control and different treatment of levels of abiotic stresses conditions in two cultivars of Hayola#50 and #4815. First, 133 BnRHs genes were identified in the rapeseed genome, which suggests that the majority role of RHs genes in modulating environmental responses. *Arabidopsis* and rice have 113 and 155 members of RH gene, respectively [46]. In our study, we are a large number of RHs genes predicted that’s close predicate to 136 members in maize and 213 members in soybean [45]. We also characterized the length of protein (aa), molecular weight (MW), isoelectric points (*p*I), and subcellular localizations of each of RHs protein identified in the genome of *Brassica napus* L. We classified RHs based on related domains into two main families *DEXDc* and *HELICc* with subfamily. In addition, most of the RHs localized in the nuclear (66%) and cytoplasm (11%), while most *DEXDC* and *HELICc* RHs proteins were predicted in the nucleus. Thus, we suggest that the RHs mainly function in the processing of RNA. In this study, Some RHs proteins were predicted in chloroplast and mitochondria with some unknown situations. In maize chloroplast, *R3H DEAD*-box [47] and *Arabidopsis* mitochondria *ABO6* RNA helicase have a function in the splicing of RNA [48]. One hundred thirty-three BnRHs genes were localized on *Brassica napus* chromosomes, as shown in Fig. 5. The results of analysis of chromosomal location demonstrated the density of distribution of RHs that ranged from 1% (chro#3) to 27% (chro#10) contained fewer and the largest number of RHs genes, respectively.

In Fig. 1C, the phylogentic analysis showed that BnRHs genes in *Brassica napus* classified into main and subgroups. However, Xu et al. [45] reported that RHs genes are classified into many more subclades of *Arabidopsis*, rice, maize, and soybean. The variety in the member and compositions of subclades from various plant species demonstrate diversity between composition in RHs genes in different plant species [43, 44]. In addition, the gene structure analysis of exon-intron and conserved domains increase knowledge evolution of gene families [45], and divergence can generate homologous genes with different functions.

*Cis*-element is the rejoin of the promoter related to the regulation of gene expression. In this study, we identified *Cis*-elements for RHs genes as having different responses to abiotic stresses, indicating the role of RHs in abiotic stresses. Several evidence has been published by several researchers indicating that DEAD-box RHs have an essential role in responses to abiotic stress in different plant species. For example, *RCF1* [49], *AtRH7* [23], *OsBIRH1*, *OsRH58*, and *OsRH42* [24, 50, 51] play important roles in abiotic stress, including drought, salinity, cold and oxidative stress tolerance and growth plants.

As advances in large-scale sequencing efforts have been made, genomic comparison approaches have been increasingly used to facilitate evolutionary and functional analysis, as conserved sequences can infer evolutionary processes. The concepts of orthology and paralogy originate from the molecular systematic domain. Orthologists and paralogues are two major types of homologs: the first type evolved through separation from a common ancestor, and the second type associated with reproductive events. Genomic comparison, the classification of orthologous genes, provides a framework for combining information from multiple genomes and highlights the divergence and conservation of gene families and biological processes. The Identification of orthology groups in prokaryotic genomes has made it possible to cross-reference genes from different species, facilitate genome annotation, classify protein families, and study bacterial evolution. The process of orthologous diagnosis, in addition to being closely related to comparative analysis and genomic dynamics, is a very important field of study to help improve the annotation of the performance of different organisms and still explaining the evolved processes is a very important species [52]. As seen in Additional file 2, the orthologous relationships between the RHs genes on chromosomes S1 and B3 are more significant than on other chromosomes, indicating the evolutionary relationships and importance of these chromosomes.

According to the gene ontology analysis, BnRHs are involved in stress-related pathways, suggesting their significance in environmental stress conditions. BnRHs played roles in binding, helicase activity, cellular component and response to salt, water deprivation, cold and cadmium (Cd) (Fig. 4). The results obtained from gene ontology analysis were found to be compatible with results of previous studies in which the relationship between BnRHs genes and several abiotic stress conditions was investigated.

We have measured the stress-related physiological indicators under control and abiotic stress growth conditions. Relative water content (RWC), Na^+^/K^+^, proline and cadmium are used as indicators to measure plant tolerance to abiotic stress in rapeseed [15, 53]. The identification of the putative RHs genes using bioinformatics approaches will provide useful information for future studies on the biological functions of the RHs gene family. To our knowledge, this is the first report on the identification and possible role of the RHs genes family in rapeseed for understanding the function of gene family in growth and environmental stress conditions response mechanism. Deciphering the mechanism of RHs in rapeseed provides different aspects for molecular breeding.

## Conclusions

We performed a comprehensive genome-wide survey of RHs in rapeseed, including phylogeny, chromosomal localization and distribution, events of duplication, gene structure, protein motif and different aspects using bioinformatics approaches and validating by greenhouse and laboratory experiments. A total of 133 BnRHs genes were identified and the level of expression 10 genes was confirmed by qRT-PCR under control and abiotic stress in two cultivars (Hayola#50 and #4815). This finding provides new insight for understanding the function and possible role of RHs mechanism in response to abiotic stress in rapeseed. The results obtained from this study show that different genotypes show different responses to some abiotic stresses under different conditions. The results show that it is not possible to introduce a single cultivar to deal with different stresses. Comparison of two rapeseed genotypes #50 and #4815 showed that two cultivars showed different reactions to abiotic stresses. Evaluation of physiological and morphological criteria, plant growth and establishment conditions in open greenhouse, despite the introduction of Hayola#50 cultivar to farmers, showed that genotype #4815 was superior to Hayola#50 and so-called tolerant genotype. Examination of the expression pattern of RHs genes identified in rapeseed also confirmed that in heat, cold, salinity and Cd stresses, the cultivar of Hayola#4815 was more tolerant in some stress levels than Hayola#50. On the other hand, in response to the drought stress, Hayola#50 has shown a relatively high expression. It is suggested that the quantity and quality of oil of these two rapeseed genotypes should also be examined. In additions, understanding the pathways of candidate genes for abiotic stresses to promote tolerance traits to stress will improve germplasm for the future. With tools such as next-generation sequencing (NGS), breeding technologies, and quantitative trait loci (QTLs), scientists have a solid foundation for understanding and improving the genetic traits of rapeseed for environmental conditions. In this way, by increasing their knowledge about these two genotypes, they can be used in breeding programs.

### Supplementary Information

**Supplementary Material 1.**

**Supplementary Material 2.**

**Supplementary Material 3.**

**Supplementary Material 4.**

**Supplementary Material 5.**

**Supplementary Material 6.**

**Supplementary Material 7.**

**Supplementary Material 8.**

## Data Availability

Nucleotide, protein and genome sequences rapeseed, Arabidopsis and *Solanum lycopersicum* that were used in this study were downloaded from NCBI database (http://www.ncbi.nlm.nih) and related database. Data sets supporting the results of this paper are included in the article and in supporting additional files.
