# Supplementary material for: Deciphering the possible role of RNA-helicase genes mechanism in response to abiotic stresses in rapeseed (Brassica napus L.)
Source: BMC Plant Biol. 2024 Mar 20;24:206. doi: 10.1186/s12870-024-04893-0 (PMC10953219; doi:10.1186/s12870-024-04893-0)
Supplement: Supplementary file 1 — Supplementary Material 1. [file 12870_2024_4893_MOESM1_ESM.docx]

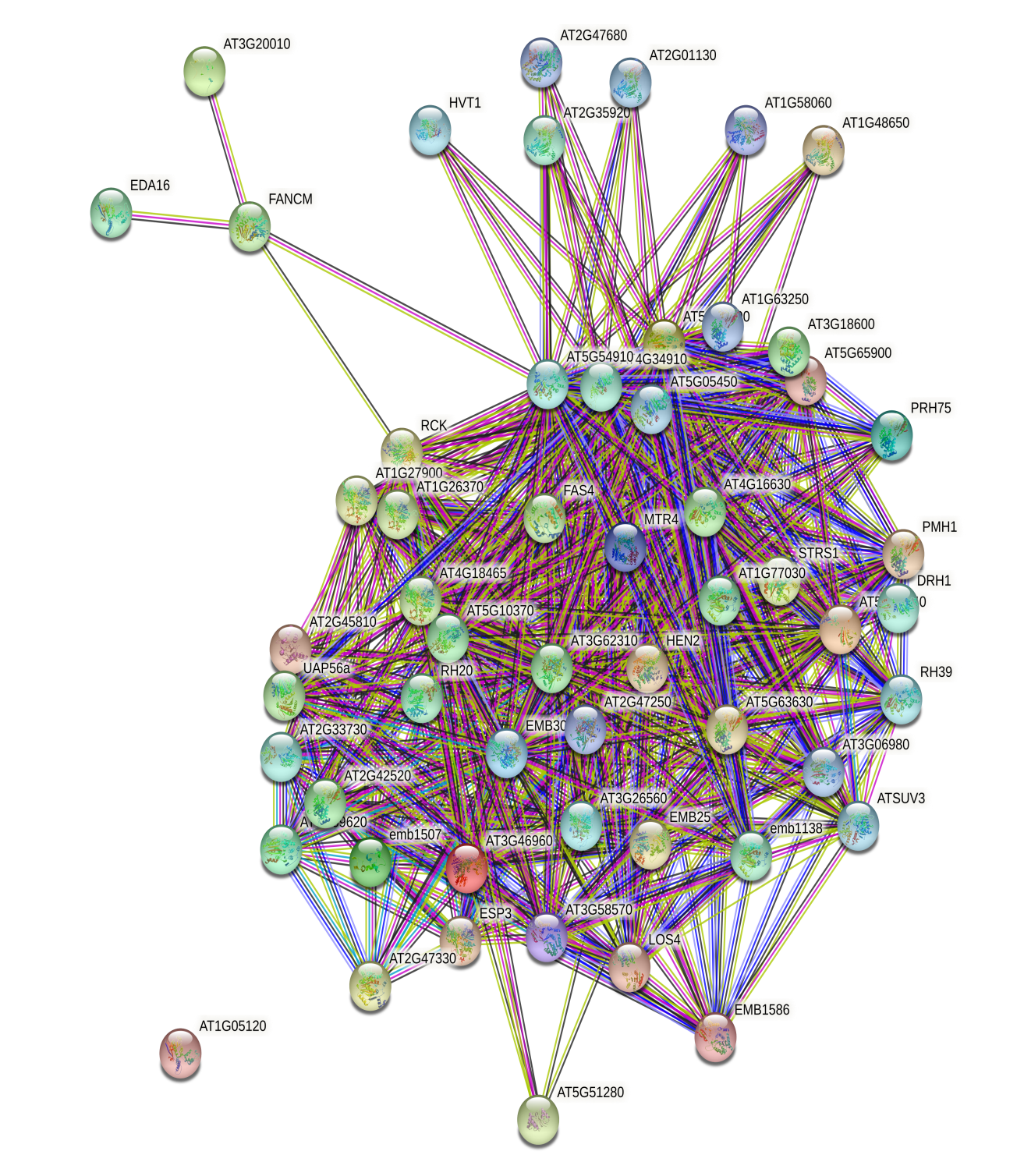

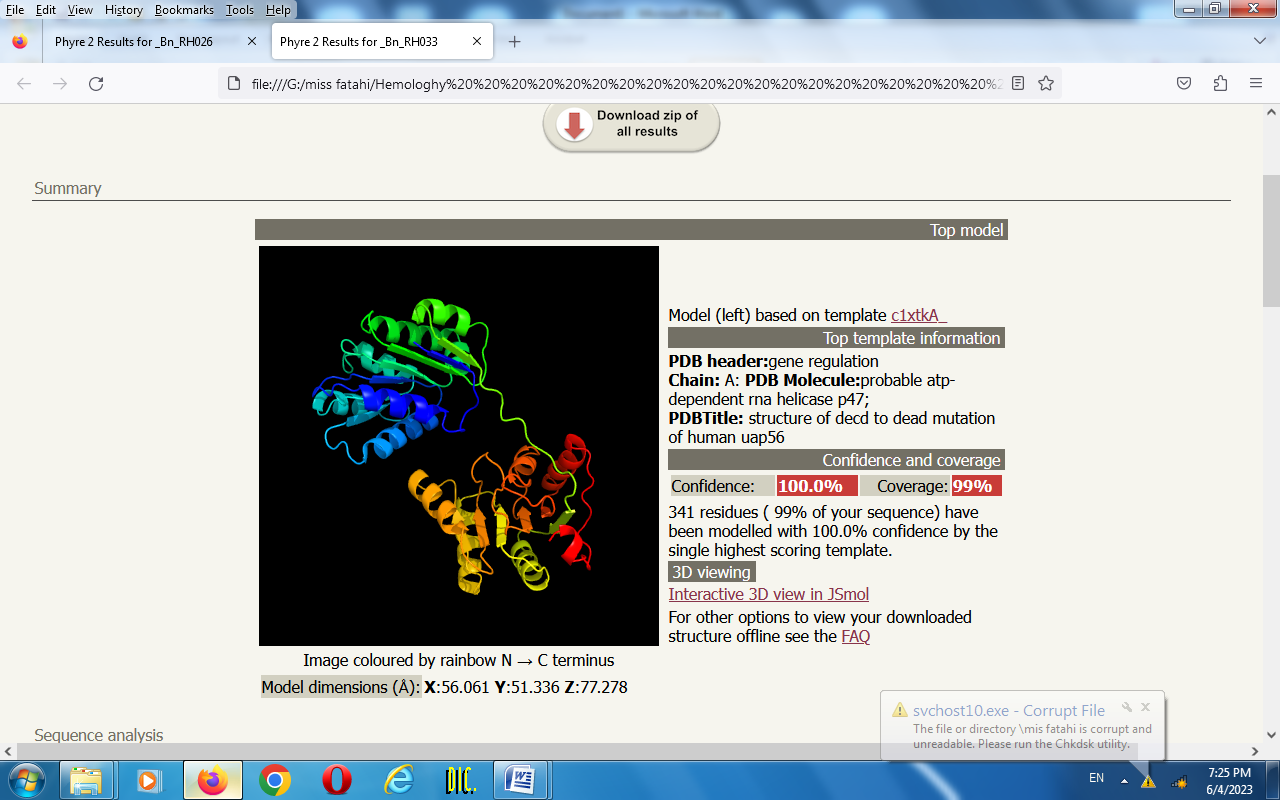

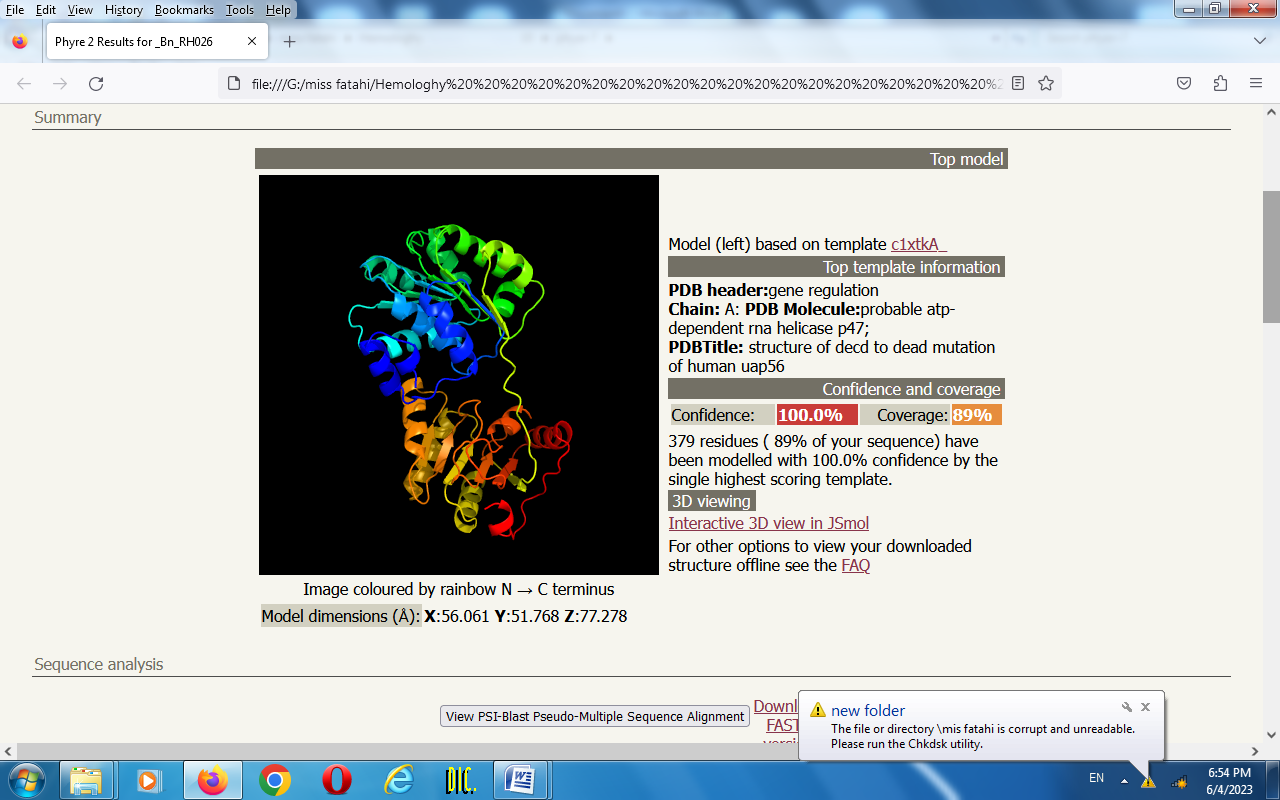

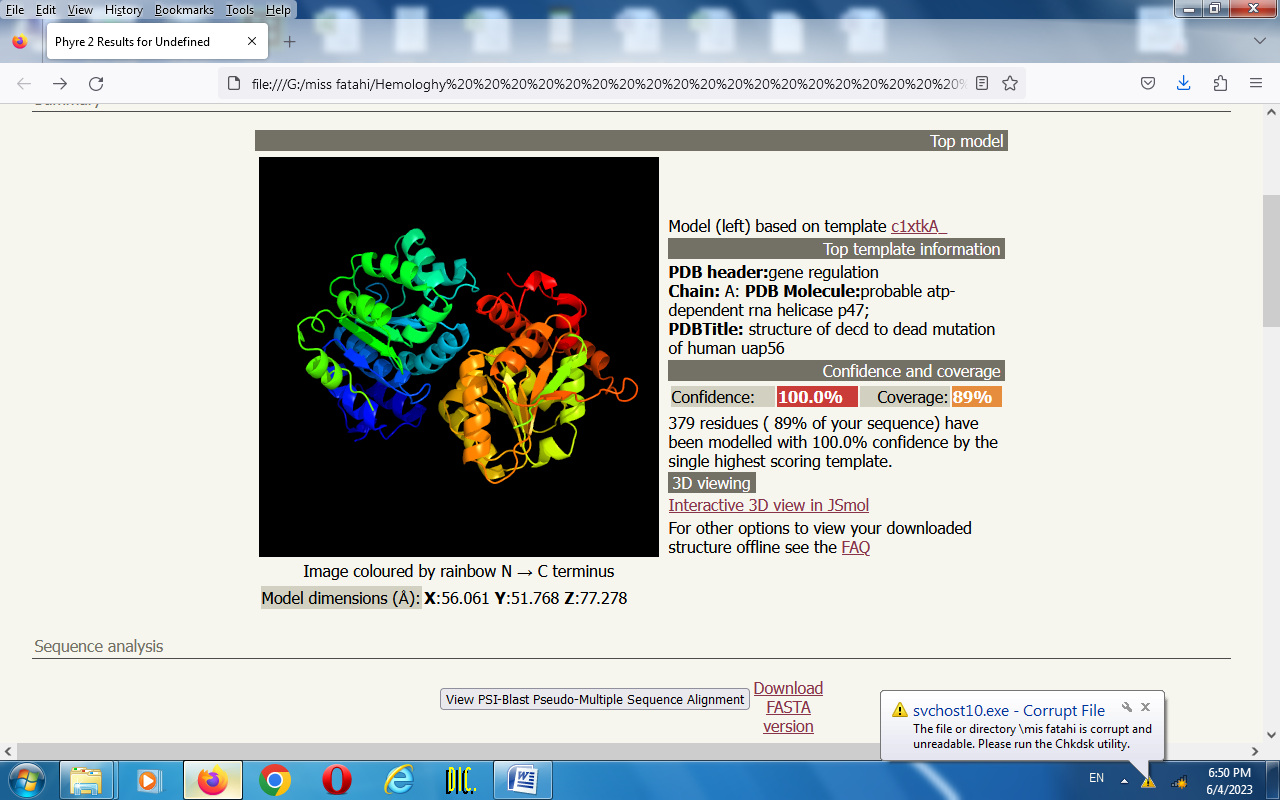


**B**

**A**

**BnRH-033**

**BnRH-026**

**BnRH-022**


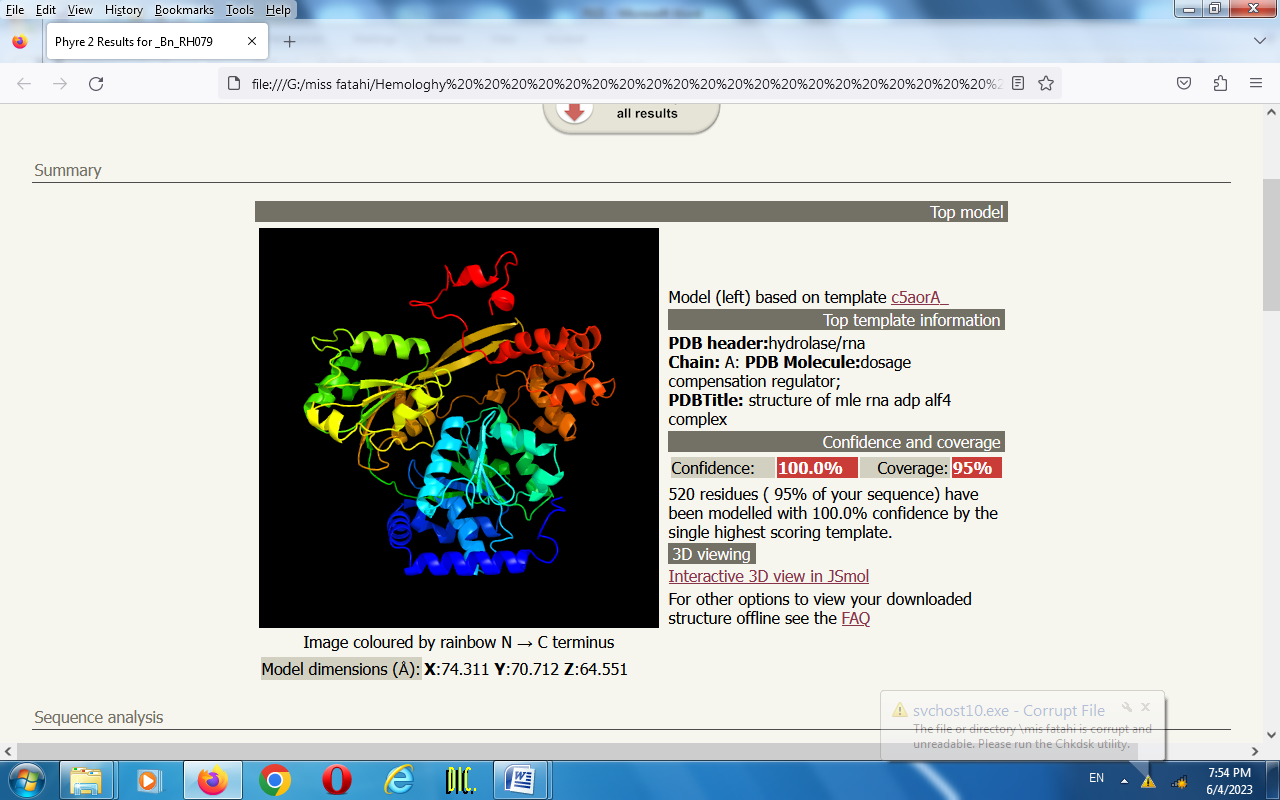

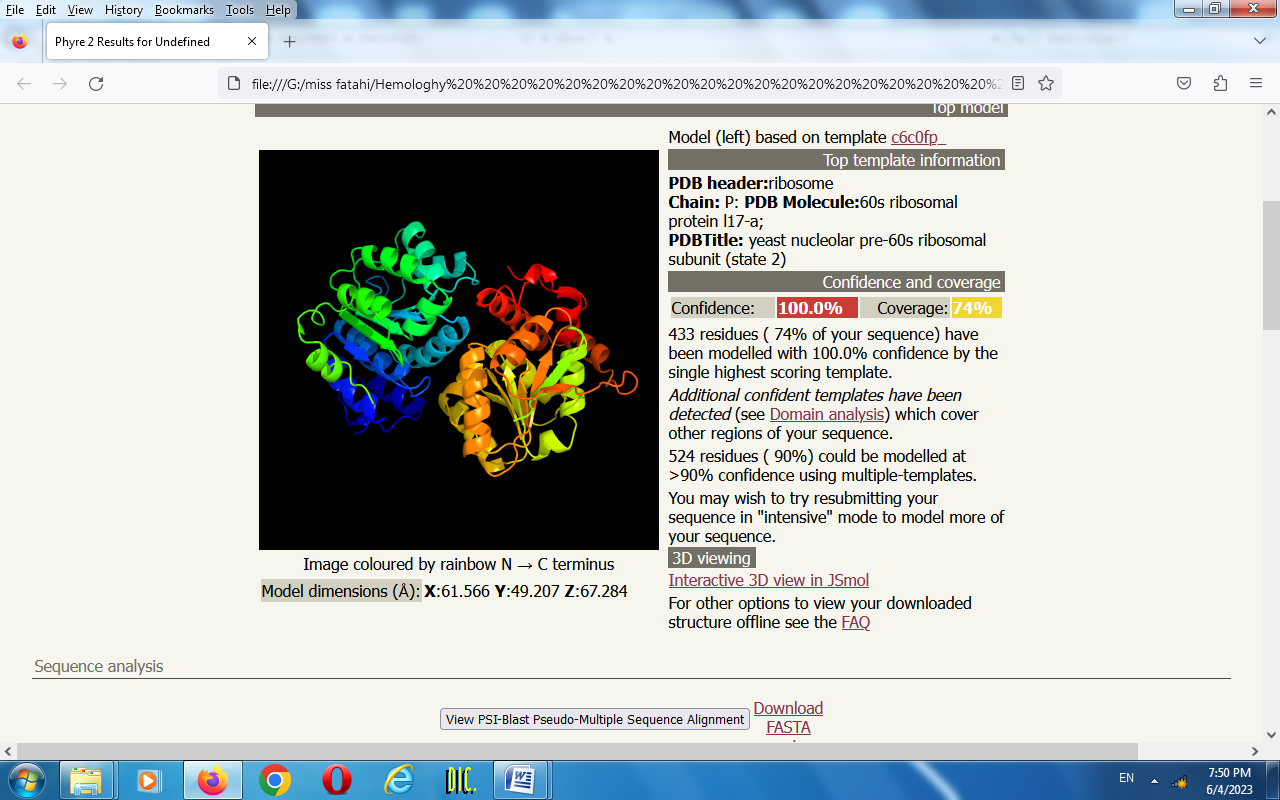

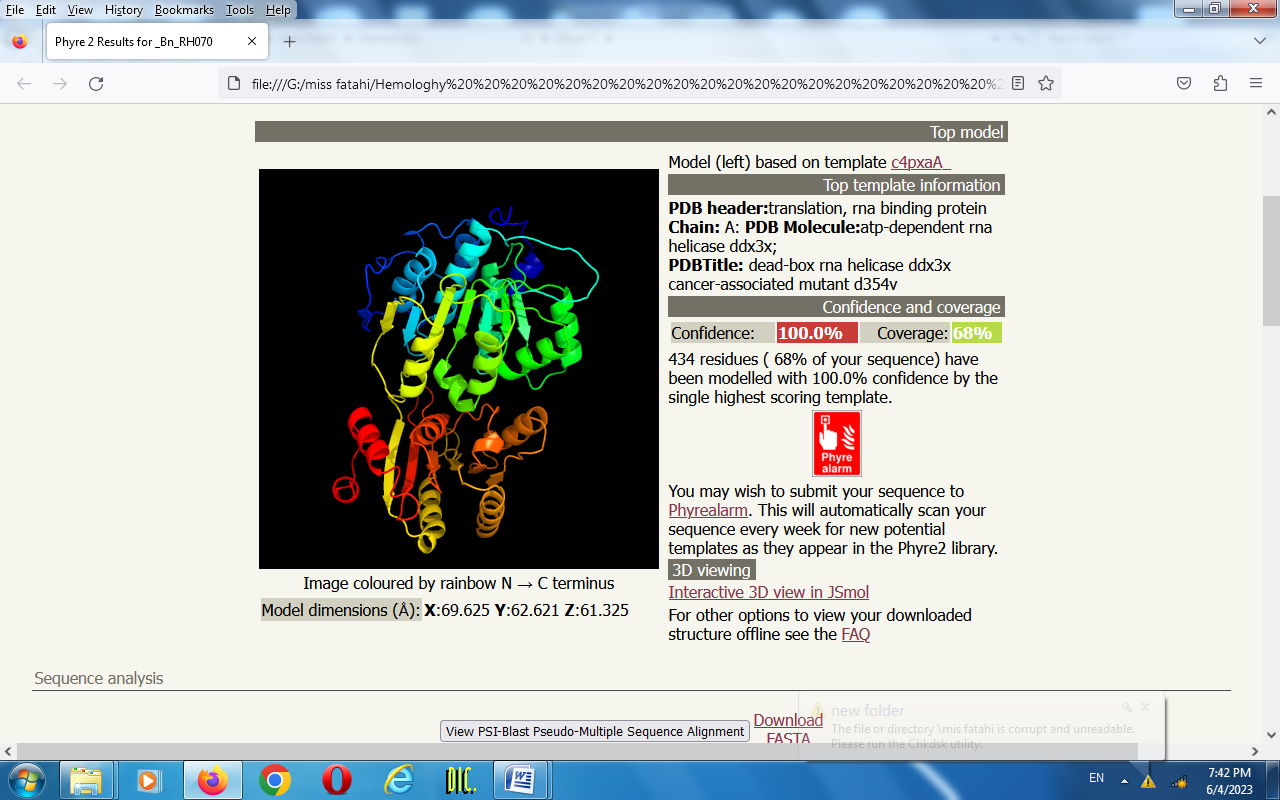


**BnRH-079**

**BnRH-025**

**BnRH-070**


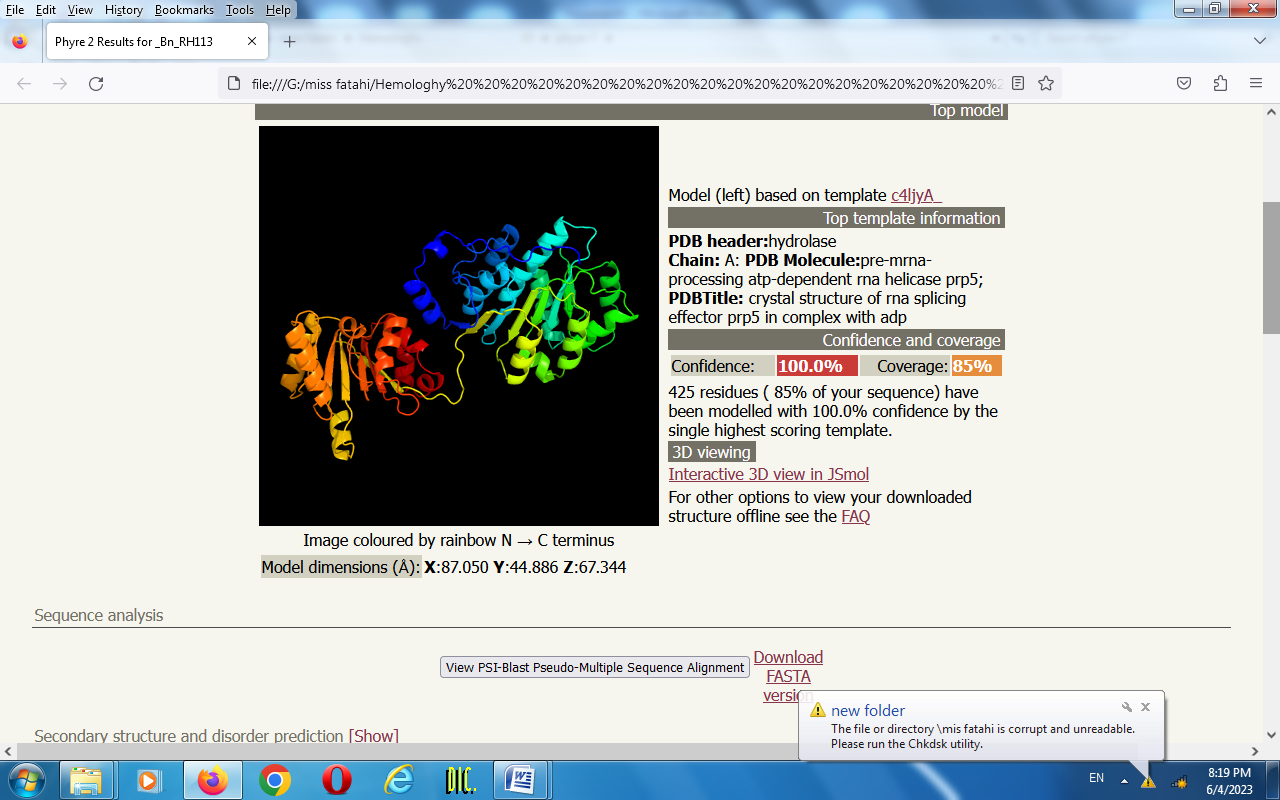

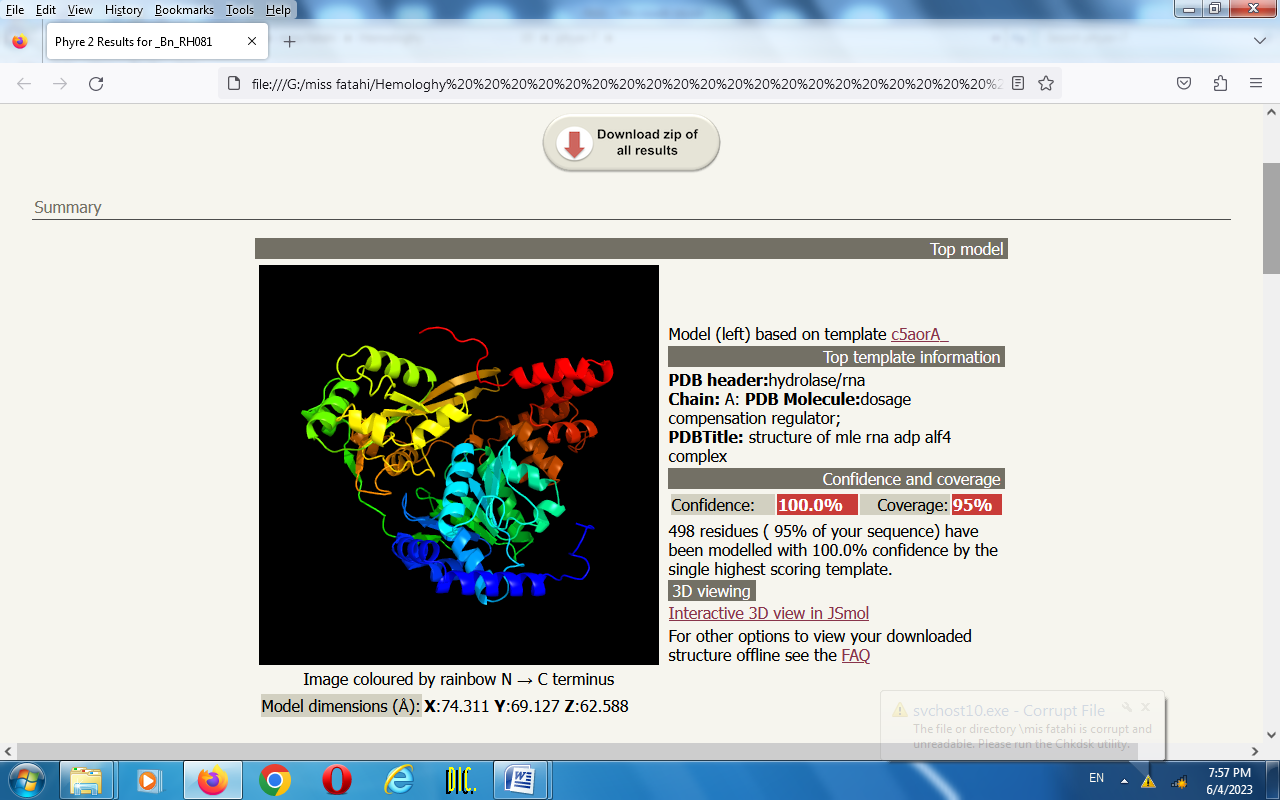


**Additional file 1.** 3D model of BnRNA-helicase proteins with 90% or more similarity (A); and protein-protein interaction of BnRHs genes network (B) in *Brassica napus* L.

**BnRH-133**

**BnRH-081**
