# Supplementary material for: Deciphering the possible role of RNA-helicase genes mechanism in response to abiotic stresses in rapeseed (Brassica napus L.)
Source: BMC Plant Biol. 2024 Mar 20;24:206. doi: 10.1186/s12870-024-04893-0 (PMC10953219; doi:10.1186/s12870-024-04893-0)
Supplement: Supplementary file 2 — Supplementary Material 2. [file 12870_2024_4893_MOESM2_ESM.docx]

**Additional file 2.** Parallel relationships between orthologous pairs of RNA helicase genes in *Brassica napus* and *Solanum lycopersicum*. Chromosomal location 0 means unknown specific gene location on *B. napus* genome. Chr., Ka, Ks and λ represents chromosome, nonsynonymous, synonymous and time duplication and divergence, respectively.

| **Gene_1** | **Chr.** | **Start 1** | **Stop 1** | **Gene_ID** | **Chr.** | **Start 2** | **Stop2** | **%identity** | **E-value** | **Ka/Ks** | **Ka** | **Ks** | **Ks/2ƛ** |
| --- | --- | --- | --- | --- | --- | --- | --- | --- | --- | --- | --- | --- | --- |
| Bn_RH010 | 6 | 420376 | 423140 | *Solanum lycopersicum*XP_004243751.1 | 7 | 16831160 | 16834939 | 80.42 | 0 | 0.9667 | 0.0589432 | 0.06097578 | 4647544.21 |
| Bn_RH014 | 7 | 3053762 | 3055444 | *Solanum lycopersicum*XP_004235903.1 | 3 | 4176856 | 4179527 | 85.03 | 0 | 1.7088 | 0.0023531 | 0.00137703 | 104956.555 |
| Bn_RH022 | 3 | 169756 | 172283 | *Solanum lycopersicum*XP_004245029.1 | 8 | 2431041 | 2436443 | 92.27 | 0 | 0.9061 | 0.0009061 | 1E-10 | 0.00762195 |
| Bn_RH025 | 3 | 169756 | 172283 | *Solanum lycopersicum*XP_004245029.1 | 8 | 2431041 | 2436443 | 92.04 | 0 | 0 | 0 | 1E-10 | 0.00762195 |
| Bn_RH026 | 3 | 169756 | 172283 | *Solanum lycopersicum*XP_004245029.1 | 8 | 2431041 | 2436443 | 92.04 | 0 | 1.5573 | 0.0083133 | 0.00533819 | 406874.238 |
| Bn_RH033 | 3 | 169756 | 172000 | *Solanum lycopersicum*XP_004245029.1 | 8 | 2431041 | 2436443 | 94.19 | 0 | 0.9789 | 0.0009937 | 0.00101507 | 77368.1402 |
| Bn_RH035 | 0 | 108823 | 111283 | *Solanum lycopersicum*XP_004248229.1 | 9 | 3482751 | 3490907 | 80.19 | 0 | 0 | 0 | 1E-10 | 0.00762195 |
| Bn_RH036 | 3 | 169756 | 172283 | *Solanum lycopersicum*XP_004241774.1 | 6 | 6430941 | 6437201 | 89.02 | 0 | 0.6168 | 0.0059474 | 0.00964216 | 734920.732 |
| Bn_RH045 | 3 | 169756 | 172000 | *Solanum lycopersicum*XP_004245029.1 | 8 | 2431041 | 2436443 | 92.73 | 0 | 0.5804 | 0.0321912 | 0.05546769 | 4227720.27 |
| Bn_RH048 | 3 | 896000 | 898879 | *Solanum lycopersicum*XP_004229376.1 | 1 | 220717 | 226110 | 87.72 | 0 | 0.8625 | 0.0090518 | 0.0104952 | 799939.024 |
| Bn_RH049 | 3 | 896000 | 898879 | *Solanum lycopersicum*XP_004229376.1 | 1 | 220717 | 226110 | 87.72 | 0 | 0.9698 | 0.0204715 | 0.0211089 | 1608910.06 |
| Bn_RH050 | 3 | 896794 | 898879 | *Solanum lycopersicum*XP_004229376.1 | 1 | 220717 | 226110 | 87.07 | 0 | 0 | 0 | 1E-10 | 0.00762195 |
| Bn_RH051 | 3 | 896803 | 898879 | *Solanum lycopersicum*XP_004229376.1 | 1 | 220717 | 226110 | 87.38 | 0 | 1.0539 | 0.2722 | 0.2583 | 19687500 |
| Bn_RH069 | 7 | 3053762 | 3054853 | *Solanum lycopersicum*XP_004235903.1 | 3 | 4176856 | 4179527 | 85.03 | 0 | 0.035 | 8.076E-05 | 0.00230919 | 176005.335 |
| Bn_RH071 | 2 | 1036267 | 1040371 | *Solanum lycopersicum*XP_004249090.1 | 10 | 2314321 | 2325222 | 85.42 | 0 | 0.7512 | 0.0503284 | 0.06699497 | 5106323.93 |
| Bn_RH079 | 8 | 240734 | 242806 | *Solanum lycopersicum*XP_004229376.1 | 1 | 220717 | 226110 | 87.14 | 0 | 1.3406 | 0.0114484 | 0.00853981 | 650900.152 |
| Bn_RH080 | 8 | 240722 | 242806 | *Solanum lycopersicum*XP_004229376.1 | 1 | 220717 | 226110 | 86.67 | 0 | 0 | 0 | 1E-10 | 0.00762195 |
| Bn_RH081 | 8 | 241092 | 242806 | *Solanum lycopersicum*XP_004229376.1 | 1 | 220717 | 226110 | 87.14 | 0 | 0.9946 | 0.0086912 | 0.00873855 | 666048.018 |
| Bn_RH099 | 3 | 1103675 | 1105270 | *Solanum lycopersicum*XP_004229376.1 | 1 | 220717 | 226110 | 84.16 | 0 | 0.9805 | 0.0179541 | 0.01831037 | 1395607.47 |
| Bn_RH119 | 3 | 1103675 | 1106068 | *Solanum lycopersicum*XP_004229376.1 | 1 | 220717 | 226110 | 85.62 | 0 | 0.8625 | 0.0090518 | 0.0104952 | 799939.024 |
| Bn_RH126 | 8 | 239870 | 242806 | *Solanum lycopersicum*XP_004229376.1 | 1 | 220717 | 226110 | 87.14 | 0 | 0.6881 | 0.0195744 | 0.02844716 | 2168228.66 |
