# Supplementary material for: Deciphering the possible role of RNA-helicase genes mechanism in response to abiotic stresses in rapeseed (Brassica napus L.)
Source: BMC Plant Biol. 2024 Mar 20;24:206. doi: 10.1186/s12870-024-04893-0 (PMC10953219; doi:10.1186/s12870-024-04893-0)
Supplement: Supplementary file 3 — Supplementary Material 3. [file 12870_2024_4893_MOESM3_ESM.docx]

**Additional file 3.** Parallel relationships between orthologous pairs of RNA helicase genes in *Brassica napus* L. and *Arabidopsis thaliana.* Chr., Ka, Ks and λ represents chromosome, nonsynonymous, synonymous and time duplication and divergence, respectively.

| **Gene_1** | **Chr.** | **Start1** | **Stop1** | **Gene_ID** | **Chr.** | **Start2** | **Stop2** | **%identity** | **E-value** | **Ka/Ks** | **Ka** | **Ks** | **Ks/2ƛ** |
| --- | --- | --- | --- | --- | --- | --- | --- | --- | --- | --- | --- | --- | --- |
| Bn_RH002 | 9 | 208559 | 211653 | *Arabidopsis thaliana* [NP_173961.3](http://www.ncbi.nlm.nih.gov/protein/NP_173961.3) | 1 | 9122030 | 9125368 | 84.1 | 0 | 0.8425 | 0.035143 | 0.0417 | 3179308 |
| Bn_RH003 | 7 | 209724 | 211043 | *Arabidopsis thaliana*  [NP_177164.1](http://www.ncbi.nlm.nih.gov/protein/NP_177164.1) | 1 | 26390016 | 26394148 | 89.68 | 0 | 0.9311 | 0.02612066 | 0.0281 | 2138139 |
| Bn_RH005 | 3 | 1494980 | 1498357 | *Arabidopsis thaliana*  [NP_190280.5](http://www.ncbi.nlm.nih.gov/protein/NP_190280.5) | 3 | 17291004 | 17298067 | 90.94 | 0 | 0.7468 | 0.02351205 | 0.0315 | 2399656 |
| Bn_RH006 | 4 | 134261 | 136180 | *Arabidopsis thaliana*  [NP_198800.1](http://www.ncbi.nlm.nih.gov/protein/NP_198800.1) | 5 | 15946769 | 15949186 | 82.59 | 0 | 1.0414 | 0.04294884 | 0.0412 | 3143278 |
| Bn_RH007 | 6 | 233177 | 235993 | *Arabidopsis thaliana*  [NP_201168.2](http://www.ncbi.nlm.nih.gov/protein/NP_201168.2) | 5 | 25472598 | 25476402 | 83.45 | 0 | 1.1967 | 0.04802919 | 0.0401 | 3058938 |
| Bn_RH008 | 4 | 541313 | 545395 | *Arabidopsis thaliana*  [NP_850154.2](http://www.ncbi.nlm.nih.gov/protein/NP_850154.2) | 2 | 13120585 | 13126635 | 87.16 | 0 | 0.8378 | 0.0362779 | 0.0433 | 3300427 |
| Bn_RH009 | 7 | 1364611 | 1367118 | *Arabidopsis thaliana*  [NP_001190402.1](http://www.ncbi.nlm.nih.gov/protein/NP_001190402.1) | 5 | 9285540 | 9288618 | 91.49 | 0 | 0.7694 | 0.02258875 | 0.0294 | 2237768 |
| Bn_RH010 | 6 | 420376 | 423140 | *Arabidopsis thaliana*  [NP_189288.1](http://www.ncbi.nlm.nih.gov/protein/NP_189288.1) | 3 | 9750122 | 9753719 | 95.79 | 0 | 1.161 | 0.02788649 | 0.024 | 1830782 |
| Bn_RH011 | 7 | 28076 | 33816 | *Arabidopsis thaliana*  [NP_178223.2](http://www.ncbi.nlm.nih.gov/protein/NP_178223.2) | 2 | 88847 | 94635 | 83.79 | 0 | 0.8765 | 0.04458945 | 0.0509 | 3877396 |
| Bn_RH012 | 1 | 146054 | 149326 | *Arabidopsis thaliana*  [NP_193396.3](http://www.ncbi.nlm.nih.gov/protein/NP_193396.3) | 4 | 93621 | 9366449 | 81.36 | 0 | 0.9424 | 0.04705617 | 0.0499 | 3805803 |
| Bn_RH014 | 7 | 3053762 | 3055444 | *Arabidopsis thaliana*  [NP_199941.1](http://www.ncbi.nlm.nih.gov/protein/NP_199941.1) | 5 | 20841456 | 20843645 | 93.3 | 0 | 0.7265 | 0.01437175 | 0.0198 | 1507822 |
| Bn_RH015 | 7 | 3053762 | 3055444 | *Arabidopsis thaliana*  [NP_199941.1](http://www.ncbi.nlm.nih.gov/protein/NP_199941.1) | 5 | 20841456 | 20843645 | 85.45 | 0 | 0.6769 | 0.01642125 | 0.0243 | 1848944 |
| Bn_RH016 | 9 | 77998 | 79107 | *Arabidopsis thaliana*  [NP_176514.1](http://www.ncbi.nlm.nih.gov/protein/NP_176514.1) | 1 | 23463284 | 23466451 | 85.06 | 0 | 0.8503 | 0.00865118 | 0.0102 | 775470.3 |
| Bn_RH018 | 10 | 19064 | 20718 | *Arabidopsis thaliana*  [NP_195217.1](http://www.ncbi.nlm.nih.gov/protein/NP_195217.1) | 4 | 16631661 | 16634834 | 82.88 | 0 | 0.8121 | 0.04537879 | 0.0559 | 4259232 |
| Bn_RH019 | 9 | 374305 | 376452 | *Arabidopsis thaliana*  [NP_201025.1](http://www.ncbi.nlm.nih.gov/protein/NP_201025.1) | 5 | 24980542 | 24983879 | 82.08 | 0 | 1.1554 | 0.08308731 | 0.0719 | 5481009 |
| Bn_RH020 | 9 | 155278 | 156587 | *Arabidopsis thaliana*  [NP_178818.1](http://www.ncbi.nlm.nih.gov/protein/NP_178818.1) | 2 | 3576483 | 3580396 | 84.33 | 0 | 1.2272 | 0.00989812 | 0.0081 | 614733.2 |
| Bn_RH021 | 9 | 295276 | 301212 | *Arabidopsis thaliana*  [NP_176103.2](http://www.ncbi.nlm.nih.gov/protein/NP_176103.2) | 1 | 21489480 | 21501775 | 87.45 | 0 | 0.7673 | 0.03920861 | 0.0511 | 3894534 |
| Bn_RH023 | 1 | 433518 | 436114 | *Arabidopsis thaliana*  [NP_193215.2](http://www.ncbi.nlm.nih.gov/protein/NP_193215.2) | 4 | 8496351 | 8499829 | 86.81 | 0 | 0.5082 | 0.0027492 | 0.0054 | 412360.5 |
| Bn_RH024 | 10 | 69125 | 70432 | *Arabidopsis thaliana*  [NP_200302.1](http://www.ncbi.nlm.nih.gov/protein/NP_200302.1) | 5 | 22298668 | 22301719 | 80.35 | 0 | 0.8377 | 0.0614274 | 0.0733 | 5588813 |
| Bn_RH026 | 3 | 169756 | 172283 | *Arabidopsis thaliana*  [NP_568245.1](http://www.ncbi.nlm.nih.gov/protein/NP_568245.1) | 5 | 3567389 | 3570686 | 96.49 | 0 | 0.5862 | 0.00851556 | 0.0145 | 1107134 |
| Bn_RH027 | 1 | 168717 | 171031 | *Arabidopsis thaliana*  [NP_193215.2](http://www.ncbi.nlm.nih.gov/protein/NP_193215.2) | 4 | 8496351 | 8499829 | 86.63 | 0 | 0 | 0 | 1E-10 | 0.007622 |
| Bn_RH028 | 1 | 11494 | 12584 | *Arabidopsis thaliana*  [NP_176185.1](http://www.ncbi.nlm.nih.gov/protein/NP_176185.1) | 1 | 21984571 | 2199011 | 88.45 | 0 | 0.7949 | 0.06611178 | 0.0832 | 6339323 |
| Bn_RH029 | 1 | 11494 | 12584 | *Arabidopsis thaliana*  [NP_176185.1](http://www.ncbi.nlm.nih.gov/protein/NP_176185.1) | 1 | 21984571 | 2179011 | 90.17 | 0 | 0.5404 | 0.00739707 | 0.0137 | 1043284 |
| Bn_RH030 | 1 | 1241658 | 1243017 | *Arabidopsis thaliana*  [NP_195217.1](http://www.ncbi.nlm.nih.gov/protein/NP_195217.1) | 4 | 16631661 | 16634834 | 83.57 | 0 | 0.9786 | 0.03991923 | 0.0408 | 3109261 |
| Bn_RH034 | 2 | 491779 | 494667 | *Arabidopsis thaliana*  [NP_196164.1](http://www.ncbi.nlm.nih.gov/protein/NP_196164.1) | 5 | 1612077 | 1615195 | 85.45 | 0 | 1.0053 | 0.04401164 | 0.0438 | 3336830 |
| Bn_RH035 | 0 | 108823 | 111283 | *Arabidopsis thaliana*  [NP_568931.1](http://www.ncbi.nlm.nih.gov/protein/NP_568931.1) | 5 | 24546601 | 24549148 | 94.12 | 0 | 0.7943 | 0.0137075 | 0.0173 | 1315391 |
| Bn_RH037 | 7 | 3665918 | 3669619 | *Arabidopsis thaliana*  [NP_177829.5](http://www.ncbi.nlm.nih.gov/protein/NP_177829.5) | 1 | 28947887 | 28951526 | 87.13 | 0 | 0.9122 | 0.03704547 | 0.0406 | 3095500 |
| Bn_RH038 | 5 | 1247232 | 1248941 | *Arabidopsis thaliana*  [NP_188490.1](http://www.ncbi.nlm.nih.gov/protein/NP_188490.1) | 3 | 6399724 | 6403007 | 85.64 | 0 | 0.6717 | 0.01367803 | 0.0204 | 1552121 |
| Bn_RH039 | 3 | 677191 | 678483 | *Arabidopsis thaliana*  [NP_180929.1](http://www.ncbi.nlm.nih.gov/protein/NP_180929.1) | 2 | 14265679 | 14267880 | 82.7 | 0 | 0.8316 | 0.0745766 | 0.0897 | 6835533 |
| Bn_RH040 | 3 | 1311916 | 1314072 | *Arabidopsis thaliana*  [NP_850255.1](http://www.ncbi.nlm.nih.gov/protein/NP_850255.1) | 2 | 15075674 | 15080506 | 92.05 | 0 | 0.8786 | 0.02309836 | 0.0263 | 2003810 |
| Bn_RH042 | 2 | 185221 | 188261 | *Arabidopsis thaliana*  [NP_189410.2](http://www.ncbi.nlm.nih.gov/protein/NP_189410.2) | 3 | 10273952 | 10280213 | 90.12 | 0 | 0.692 | 0.02579511 | 0.0373 | 2841087 |
| Bn_RH043 | 6 | 267642 | 269840 | *Arabidopsis thaliana*  [NP_190879.1](http://www.ncbi.nlm.nih.gov/protein/NP_190879.1) | 3 | 19687968 | 19690423 | 86.75 | 0 | 0.8085 | 0.03520697 | 0.0435 | 3318883 |
| Bn_RH045 | 3 | 169756 | 172000 | *Arabidopsis thaliana*  [NP_850807.2](http://www.ncbi.nlm.nih.gov/protein/NP_850807.2) | 5 | 3554272 | 3556646 | 96.22 | 0 | 1.0619 | 0.0102848 | 0.0097 | 738202.7 |
| Bn_RH048 | 3 | 896000 | 898879 | *Arabidopsis thaliana*  [NP_182247.1](http://www.ncbi.nlm.nih.gov/protein/NP_182247.1) | 2 | 19399923 | 19402981 | 94.65 | 0 | 0.8004 | 0.02519588 | 0.0315 | 2399286 |
| Bn_RH049 | 3 | 896000 | 898879 | *Arabidopsis thaliana*  [NP_182247.1](http://www.ncbi.nlm.nih.gov/protein/NP_182247.1) | 2 | 19399923 | 19402981 | 94.65 | 0 | 0.5516 | 0.00388903 | 0.0071 | 537378.8 |
| Bn_RH050 | 3 | 896794 | 898879 | *Arabidopsis thaliana*  [NP_182247.1](http://www.ncbi.nlm.nih.gov/protein/NP_182247.1) | 2 | 19399923 | 19402981 | 93.96 | 0 | 0 | 0 | 1E-10 | 0.007622 |
| Bn_RH051 | 3 | 896803 | 898879 | *Arabidopsis thaliana*  [NP_182247.1](http://www.ncbi.nlm.nih.gov/protein/NP_182247.1) | 2 | 19399923 | 19402981 | 94.31 | 0 | 0.8004 | 0.02519588 | 0.0315 | 2399286 |
| Bn_RH052 | 3 | 229605 | 232399 | *Arabidopsis thaliana*  [NP_849348.1](http://www.ncbi.nlm.nih.gov/protein/NP_849348.1) | 4 | 6136333 | 6139510 | 87.36 | 0 | 0.8072 | 0.03575993 | 0.0443 | 3376758 |
| Bn_RH057 | 3 | 153299 | 156485 | *Arabidopsis thaliana*  [NP_564296.1](http://www.ncbi.nlm.nih.gov/protein/NP_564296.1) | 1 | 9715615 | 9720346 | 89.35 | 0 | 0.8142 | 0.1187 | 0.1458 | 11112805 |
| Bn_RH058 | 3 | 153299 | 156485 | *Arabidopsis thaliana*  [NP_564296.1](http://www.ncbi.nlm.nih.gov/protein/NP_564296.1) | 1 | 9715615 | 9720346 | 89.35 | 0 | 0 | 0 | 1E-10 | 0.007622 |
| Bn_RH063 | 4 | 267662 | 269741 | *Arabidopsis thaliana*  [NP_182105.1](http://www.ncbi.nlm.nih.gov/protein/NP_182105.1) | 2 | 18859836 | 18862318 | 89.56 | 0 | 1.0176 | 0.02995684 | 0.0294 | 2243738 |
| Bn_RH101 | 3 | 677191 | 678483 | *Arabidopsis thaliana*  [NP_180929.1](http://www.ncbi.nlm.nih.gov/protein/NP_180929.1) | 2 | 14265679 | 14267880 | 81.83 | 0 | 0.8083 | 0.07770899 | 0.0961 | 7327322 |
| Bn_RH103 | 4 | 68703 | 70360 | *Arabidopsis thaliana*  [NP_181780.1](http://www.ncbi.nlm.nih.gov/protein/NP_181780.1) | 2 | 17705382 | 17708744 | 89.61 | 0 | 0.7501 | 0.01976744 | 0.0264 | 2008586 |
| Bn_RH105 | 5 | 200412 | 202272 | *Arabidopsis thaliana*  [NP_565338.1](http://www.ncbi.nlm.nih.gov/protein/NP_565338.1) | 2 | 2895135 | 2900909 | 89.98 | 0 | 1.1286 | 0.04825225 | 0.0428 | 3258815 |
| Bn_RH107 | 5 | 200412 | 202272 | *Arabidopsis thaliana*  [NP_565338.1](http://www.ncbi.nlm.nih.gov/protein/NP_565338.1) | 2 | 2895135 | 2900909 | 89.98 | 0 | 1.0571 | 0.04575814 | 0.0433 | 3299407 |
| Bn_RH112 | 9 | 1343959 | 1345272 | *Arabidopsis thaliana*  [NP_201025.1](http://www.ncbi.nlm.nih.gov/protein/NP_201025.1) | 5 | 24980542 | 24983879 | 81.78 | 0 | 0.8545 | 0.05578867 | 0.0653 | 4976406 |
| Bn_RH113 | 3 | 729532 | 732365 | *Arabidopsis thaliana*  [NP_175911.1](http://www.ncbi.nlm.nih.gov/protein/NP_175911.1) | 1 | 20574634 | 20577141 | 93.61 | 0 | 0.6767 | 0.01825339 | 0.027 | 2055848 |
| Bn_RH114 | 1 | 472691 | 474941 | *Arabidopsis thaliana*  [NP_567558.2](http://www.ncbi.nlm.nih.gov/protein/NP_567558.2) | 4 | 10197056 | 10201611 | 94.07 | 0 | 0.6303 | 0.01544282 | 0.0245 | 1867563 |
| Bn_RH121 | 5 | 11924 | 14339 | *Arabidopsis thaliana*  [NP_174479.1](http://www.ncbi.nlm.nih.gov/protein/NP_174479.1) | 1 | 11479921 | 11482707 | 86.51 | 0 | 0 | 0 | 1E-10 | 0.007622 |
| Bn_RH123 | 5 | 2024226 | 2028422 | *Arabidopsis thaliana*  [NP_182290.1](http://www.ncbi.nlm.nih.gov/protein/NP_182290.1) | 2 | 19545828 | 19550871 | 86.79 | 0 | 0.7405 | 0.03060701 | 0.0413 | 3150475 |
| Bn_RH124 | 5 | 2024226 | 2028422 | *Arabidopsis thaliana*  [NP_182290.1](http://www.ncbi.nlm.nih.gov/protein/NP_182290.1) | 2 | 19545828 | 19550871 | 87.05 | 0 | 0.7405 | 0.03060701 | 0.0413 | 3150475 |
| Bn_RH125 | 4 | 312782 | 314721 | *Arabidopsis thaliana*  [NP_566099.1](http://www.ncbi.nlm.nih.gov/protein/NP_566099.1) | 2 | 19429083 | 19431617 | 88.2 | 0 | 0.9831 | 0.02804245 | 0.0285 | 2174161 |
| Bn_RH126 | 8 | 239870 | 242806 | *Arabidopsis thaliana*  [NP_191790.1](http://www.ncbi.nlm.nih.gov/protein/NP_191790.1) | 3 | 23057516 | 23060561 | 93.53 | 0 | 0.799 | 0.00887961 | 0.0111 | 847027.4 |
| Bn_RH127 | 6 | 267642 | 269831 | *Arabidopsis thaliana*  [NP_190879.1](http://www.ncbi.nlm.nih.gov/protein/NP_190879.1) | 3 | 19687968 | 19690423 | 87.53 | 0 | 0.8202 | 0.03325726 | 0.0405 | 3090428 |
| Bn_RH129 | 7 | 209724 | 211043 | *Arabidopsis thaliana*  [NP_177164.1](http://www.ncbi.nlm.nih.gov/protein/NP_177164.1) | 1 | 26390016 | 26394148 | 89.68 | 0 | 1.0631 | 0.02720763 | 0.0256 | 1950575 |
| Bn_RH130 | 5 | 451258 | 452136 | *Arabidopsis thaliana*  [NP_001077571.1](http://www.ncbi.nlm.nih.gov/protein/NP_001077571.1) | 1 | 7286356 | 7288842 | 88.21 | 0 | 0.7249 | 0.03352489 | 0.0462 | 3525116 |
| Bn_RH131 | 5 | 451258 | 452136 | *Arabidopsis thaliana*  [NP_173516.1](http://www.ncbi.nlm.nih.gov/protein/NP_173516.1) | 1 | 7286356 | 7288842 | 87.62 | 0 | 0.9778 | 0.4923 | 0.5035 | 38376524 |
| Bn_RH133 | 4 | 541313 | 545395 | *Arabidopsis thaliana*  [NP_850154.2](http://www.ncbi.nlm.nih.gov/protein/NP_850154.2) | 2 | 13120585 | 13126635 | 87.16 | 0 | 0.7402 | 0.03149342 | 0.0425 | 3242926 |
