# Supplementary material for: Deciphering the possible role of RNA-helicase genes mechanism in response to abiotic stresses in rapeseed (Brassica napus L.)
Source: BMC Plant Biol. 2024 Mar 20;24:206. doi: 10.1186/s12870-024-04893-0 (PMC10953219; doi:10.1186/s12870-024-04893-0)
Supplement: Supplementary file 4 — Supplementary Material 4. [file 12870_2024_4893_MOESM4_ESM.docx]

**Additional file 4**. The summary of *Cis-*elements in promoter regions of identified RNA helicase genes in drought (A); salt (B); cold (C) and heat (D) stresses

**A: drought stress**

| *Cis*-elements of sequence | *Cis*-elements | number |
| --- | --- | --- |
| **CCACGTGG** | S000133 | 0 |
| **CCGAC** | S000153 | 296 |
| **CACATG** | S000174 | 160 |
| **CTAACCA** | S000175 | 0 |
| **CNGTTR** | S000176 | 452 |
| **TAACTG** | S000177 | 26 |
| **ACCGAC** | S000402 | 44 |
| **WAACCA** | S000408 | 343 |
| **CATGTG** | S000413 | 160 |
| **ACGTG** | S000414 | 230 |
| **ACGTG** | S000415 | 460 |
| **RCCGAC** | S000418 | 162 |
| **RYCGAC** | S000497 | 162 |

**B: salt stress**

| *Cis* elements of sequence | *Cis* elements | number |
| --- | --- | --- |
| **GAAAAA** | S000453 | 1190 |
| **ACCGAC** | S000402 | 44 |
| **RCCGAC** | S000418 | 162 |

**C: Cold stress**

| *Cis* elements of sequence | *Cis* elements | number |
| --- | --- | --- |
| **ACCGAC** | S000402 | 44 |
| **CANNTG** | S000407 | 2594 |
| **RCCGAC** | S000418 | 162 |

**D: Heat stress**

| *Cis* elements of sequence | *Cis* elements | number |
| --- | --- | --- |
| **CCAAT** | S000030 | 341 |
| **RCCGAC** | S000418 | 162 |
