# Supplementary material for: Deciphering the possible role of RNA-helicase genes mechanism in response to abiotic stresses in rapeseed (Brassica napus L.)
Source: BMC Plant Biol. 2024 Mar 20;24:206. doi: 10.1186/s12870-024-04893-0 (PMC10953219; doi:10.1186/s12870-024-04893-0)
Supplement: Supplementary file 5 — Supplementary Material 5. [file 12870_2024_4893_MOESM5_ESM.docx]

**Additional file 5.** Analysis of variance relative water content (RWC) in response to drought stress

| Mean of square | df | S. O.V |
| --- | --- | --- |
| RWC |  |  |
| 210.39**^**^** | 2 | Drought level |
| 72.00**^*^** | 1 | Cultivar |
| 36.17ns | 2 | Drought level × cultivar |
| 14.17 | 12 | error |
| 7.15 | - | CV |

*, **, ns indicate a significant and non-significant difference at the 1 and 5% probability level, respectively.
