# Supplementary material for: Deciphering the possible role of RNA-helicase genes mechanism in response to abiotic stresses in rapeseed (Brassica napus L.)
Source: BMC Plant Biol. 2024 Mar 20;24:206. doi: 10.1186/s12870-024-04893-0 (PMC10953219; doi:10.1186/s12870-024-04893-0)
Supplement: Supplementary file 6 — Supplementary Material 6. [file 12870_2024_4893_MOESM6_ESM.docx]

**Additional file 6.** Analysis of variance of sodium (Na) and potassium (K) of leaves and roots of Hayola#50 and #4815 *Brassica napus* L. in response to salt stress

| S.O.V | df | Mean of square | | | |
| --- | --- | --- | --- | --- | --- |
|  |  | Leaf of Na | Root of Na | Leaf of K | Root of K |
| Salt level | 2 | 43.86**^**^** | 21.91**^**^** | 627.08**^**^** | 603.25**^**^** |
| cultivar | 1 | 2.25**^**^** | 1.92**^**^** | 40.33**^**^** | 36.75**^**^** |
| Salt × cultivar | 2 | .081**^**^** | 7.58**^**^** | 1.58**^**^** | 3.25**^*^** |
| Error | 6 | 0.05 | 0.47 | 0.17 | 0.41 |
| CV | - | 4.52 | 5.79 | 0.55 | 0.86 |

*, ** indicate a significant difference at the 1 and 5% probability level, respectively.
