# Supplementary material for: Deciphering the possible role of RNA-helicase genes mechanism in response to abiotic stresses in rapeseed (Brassica napus L.)
Source: BMC Plant Biol. 2024 Mar 20;24:206. doi: 10.1186/s12870-024-04893-0 (PMC10953219; doi:10.1186/s12870-024-04893-0)
Supplement: Supplementary file 7 — Supplementary Material 7. [file 12870_2024_4893_MOESM7_ESM.docx]

**Additional file 7.** Analysis of variance leaf and root proline in Hayola#50 and #4815 rapeseed in response to salt, drought (A) and cold (B) stress.

**A:**

| Mean of square | | | | df | S .O. V |
| --- | --- | --- | --- | --- | --- |
| Drought stress | | Salt stress | |  |  |
| Root Proline | Leaf Proline | Root Proline | Leaf Proline |  |  |
| 0.0266**^**^** | 0.024**^**^** | 0.0042**^**^** | 0.010**^**^** | 2 | Salt/drought level |
| 0.0002^ns^ | 0.016**^**^** | 0.0056**^**^** | 0.004**^**^** | 1 | Cultivar |
| 0.0010^ns^ | 0.002**^*^** | 0.0006^ns^ | 0.004**^**^** | 2 | Salt/drought × cultivar |
| 0.0021 | 0.002 | 0.001 | 0.001 | 6 | Error |
| 8.77 | 6.67 | 13.13 | 6.32 | - | CV |

*, **, ns indicate a significant and non-significant difference at the 1 and 5% probability level, respectively.

**B:**

| Mean of square | | df | S . O. V |
| --- | --- | --- | --- |
| Root Proline | Leaf Proline |  |  |
| 0.0002^ns^ | 0.0113**^**^** | 4 | Cold level |
| 0.0042**^*^** | 0.0540**^**^** | 1 | cultivar |
| 0.0004^ns^ | 0.0018**^**^** | 4 | Cold level × cultivar |
| 0.0008 | 0.0004 | 10 | error |
| 12.02 | 5.40 | - | CV |

*, **, ns indicate a significant and non-significant difference at the 1 and 5% probability level, respectively.
