# Supplementary material for: Deciphering the possible role of RNA-helicase genes mechanism in response to abiotic stresses in rapeseed (Brassica napus L.)
Source: BMC Plant Biol. 2024 Mar 20;24:206. doi: 10.1186/s12870-024-04893-0 (PMC10953219; doi:10.1186/s12870-024-04893-0)
Supplement: Supplementary file 8 — Supplementary Material 8. [file 12870_2024_4893_MOESM8_ESM.docx]

**Additional file 8.** Analysis of variance cadmium and proline in response to cadmium (Cd) and heat stress

| Mean of square | | | | | | df | S .O. V |
| --- | --- | --- | --- | --- | --- | --- | --- |
| Heat stress | | Cd stress | | Cd stress | |  |  |
| Root Proline | Leaf Proline | Root Proline | Leaf Proline | Root Cd | Leaf Cd |  |  |
| 0.0017**^**^** | 0.0333**^**^** | 0.0007**^**^** | 0.0231**^**^** | 312.50**^**^** | 258.92**^**^** | 3 | Cd / heat level |
| 0.0001^ns^ | 0.0232**^**^** | 0.0004^ns^ | 0.0064**^**^** | 20.25**^*^** | 2.25**^**^** | 1 | Cultivar |
| 0.0001^ns^ | 0.0022**^*^** | 0.0013**^**^** | 0.0035**^**^** | 13.42***** | 8.92**^**^** | 3 | Cd / heat × cultivar |
| 0.0002 | 0.0004 | 0.0002 | 0.0005 | 3.25 | 3.00 | 8 | error |
| 14.17 | 8.59 | 10.71 | 11.09 | 1.23 | 3.58 |  | CV |

*, **, ns indicate a significant and non-significant difference at the 1 and 5% probability level, respectively.
